# Biological sex differences after high and low doses of influenza A virus infection during obesity

**DOI:** 10.3389/fimmu.2026.1788970

**Published:** 2026-04-10

**Authors:** Saurav Pantha, Brian Wolfe, Saranya Vijayakumar, Shristy Budha Magar, Tawfik Aboellail, Santosh Dhakal

**Affiliations:** 1 Department of Diagnostic Medicine/Pathobiology (DMP), College of Veterinary Medicine (CVM), Kansas State University (KSU), Manhattan, KS, United States; 2 Kansas State Veterinary Diagnostic Laboratory (KSVDL), College of Veterinary Medicine (CVM), Kansas State University (KSU), Manhattan, KS, United States

**Keywords:** cytokine and chemokine influx, hyperglycemia, immune dysregulation, myeloid cells, pulmonary inflammation

## Abstract

**Introduction:**

Obesity is an independent risk factor for influenza A virus (IAV) pathogenesis. In non-obese hosts, following IAV infection, adult females develop more severe disease than males. Limited research on biological sex differences following IAV infection in individuals with obesity forms the basis for this study.

**Materials and methods:**

Male and female C57BL/6J mice were treated with high-fat or low-fat diets to obtain mice with or without obesity. They were inoculated intranasally with a high (10^3^) or low dose (10^1.5^ TCID_50_) of mouse-adapted A/California/04/2009 H1N1 IAV and followed for 21 days post-infection (dpi) for morbidity. Subsets of mice were euthanized at different dpi to measure lung virus titers, histopathological changes, cellular infiltration, and cytokine induction.

**Results:**

After a high-dose infection, females with obesity demonstrated shorter median survival time, greater parenchymal inflammation, and heightened induction of inflammatory markers than males with obesity. After a low-dose infection, females more than males, and mice with obesity than the non-obese mice, exhibited severe morbidity. Female mice with obesity exhibited the greatest disease severity, where 25% (2/8) reached humane endpoint. Delayed but persistent inflammatory changes were observed in females with obesity, characterized by relatively lower pathological changes, lesser induction of cytokines and chemokines, and reduced myeloid and lymphoid infiltration in the lungs at 3 dpi, followed by sustained pathological changes and cytokine and chemokine induction up to 21 dpi.

**Discussion:**

Our study suggests that the biological sex difference following IAV infection is observed during obesity. Females with obesity experience greater influenza disease severity than males, specially following a low-dose IAV infection, possibly driven by more severe dysregulation of inflammatory responses.

## 1 Introduction

Obesity (i.e., having a body mass index, BMI ≥30) is characterized by excessive fat deposition and a state of chronic low-grade inflammation in the body. Since the 1990s, obesity has doubled in adults and quadrupled among children and adolescents, and more than 1 billion people in the world are living with obesity (1). By 2035, 51% of the world’s population is estimated to be either overweight or obese (2). In the US, 2 in 5 adults were living with obesity in 2023, and it is estimated that by 2050, at least 1 in 5 children (5–14 years), 1 in 3 adolescents (15–24 years), and 2 in 3 adults (≥25 years) will have obesity (3, 4). Increased consumption of ultra-processed foods is a major dietary factor driving the global rise in obesity (5).

Influenza viruses cause over a billion cases of respiratory illnesses annually, with 3–5 million severe cases and up to 650,000 deaths (6). Obesity was recognized as a risk factor for severe outcomes of influenza, including increased hospitalizations, the need for intensive care, and deaths during the 2009 H1N1 influenza A virus (IAV) pandemic (7). This has been observed subsequently, even during seasonal influenza virus outbreaks, further confirming obesity as an independent risk factor for influenza-associated disease severity (8, 9). A systematic review and meta-analysis showed that people with obesity have a 56% and 100% higher risk of severe disease and deaths associated with influenza, respectively, compared to people without obesity (8). Symptomatic adults with obesity are likely to shed IAV 42% longer than non-obese adults, while asymptomatic adults with obesity shed IAV 104% longer (10). Human and animal model studies have shown that obesity is associated with delayed virus clearance, progression to viral pneumonia, emergence of IAV strains, and a higher risk of secondary bacterial infections (11).

Biological sex (i.e., being male or female based on chromosomal and hormonal differences) has an important role in IAV pathogenesis and immunity. Epidemiological data in humans show that adult females of reproductive age are more likely to experience higher influenza-related hospitalization rates than their age-matched males (12, 13). Analysis of age-standardized notification rates of laboratory-confirmed cases of influenza between 2009 and 2015 in Australia showed significantly higher cases in adult females for both the H1N1 and H3N2 IAVs compared to adult males, suggesting that females are more susceptible to IAVs (14). The IAV challenge study in humans also showed that adult females had more symptoms and a longer duration of symptoms than males (15). The non-obese adult mouse model of IAV infection shows that females suffer with more severe outcome from IAV infection than males, associated with elevated inflammatory immune responses and pulmonary tissue damage, but comparable pulmonary virus replication (16, 17). In mouse models, elevated levels of testosterone and amphiregulin (i.e., a protein that drives lung tissue repair) in adult males were associated with better influenza outcomes, while administration of high doses of estradiol reduced the severe outcomes in females (16, 17), indicating the roles of sex steroids in mediating sex differences in non-obese hosts.

Sex differences exist in adiposity, adipose tissue inflammation, and innate and adaptive immunity, resulting in differential outcomes of viral infection, vaccine responses, and cardiometabolic diseases during obesity (18). Our recent study suggests that, in a mouse model of high-fat diet-induced obesity (DIO), females develop greater antibody responses and are better protected than males with obesity (19). Although sex differences during IAV infection have been studied in non-obese hosts, such studies remain limited during obesity. In this study, we used a DIO mouse model to investigate biological sex differences in virus replication, pulmonary inflammation, and disease severity following infection with both high- and low-dose 2009 pandemic H1N1 IAV. IAV-infected mice were followed for morbidity measurements up to 21 dpi, with subsets of mice euthanized at different dpi to compare temporal changes in influenza pathogenesis. Our data suggests that biological sex impacts IAV pathogenesis, where females with obesity suffer from more severe disease compared to males.

## 2 Materials and methods

### 2.1 Animals and diet treatment

All animal procedures were carried out in accordance with the Institutional Animal Care and Use Committee-approved protocol (4855) at Kansas State University (KSU). Male and female C57BL/6J mice (strain 000664, Jackson Laboratory, Bar Harbor, ME, USA) were purchased at 4–5 weeks of age and after a week of acclimatization, randomly assigned either to a low-fat diet (LFD, 10 kCal%, D12450Ji, Research Diets, New Brunswick, NJ, USA) or a high-fat diet (HFD, 60 kCal%, D12492i, Research Diets, New Brunswick, NJ, USA) treatments. Each week, body mass was measured, and after 13 to 14 weeks, mice on HFD that gained ≥20% body mass compared to the average body mass of age- and sex-matched mice on LFD were defined as mice with obesity (20). Those on HFD but did not reach the obesity threshold were defined as non-responders. This was observed among females, and non-responders were excluded from the analysis comparing mice with or without obesity. Data of non-responder females was compared with that of responder females (i.e., with obesity) in section 3.9.

### 2.2 Glucose tolerance test

GTT was performed on the 13^th^ or 14^th^ week of diet treatment. For GTT, after 6 hours of fasting, a 25% glucose solution was administered intraperitoneally (dose: 2 g/Kg body mass). Blood glucose levels were measured at different time points, including before fasting (i.e., to measure the fed glucose), at 0 min (i.e., after fasting, and immediately before glucose administration to measure fasting glucose), and at 15, 30, 60, or 120 minutes after glucose administration using the AlphaTrak3 blood glucose monitoring system (Zoetis, NJ, USA) by pricking the tip of tail with a sterile lancet (20).

### 2.3 Measurement of different metabolic markers

Plasma concentrations of leptin (#90030, Crystal Chem, IL, USA); adiponectin (#80569, Crystal Chem, IL, USA); and total cholesterol (#STA-384; Cell Biolabs Inc., CA, USA) were measured following the manufacturer’s instructions, at week 0 (i.e., before diet treatment) and after 14 weeks of diet treatment.

### 2.4 Virus infection and post-infection monitoring

The mouse-adapted A/California/04/2009 H1N1 IAVs grown in Madin-Darby Canine Kidney (MDCK) cells were used for virus infection after 13 or 14 weeks of diet treatment. For high-dose and low-dose infection studies, 10^3^ TCID_50_ and 10^1.5^ TCID_50_ doses were used, which represent lethal and sublethal doses, respectively, in non-obese adult mice (21). Viruses were diluted in Dulbecco’s Modified Eagle Medium (DMEM) and delivered intranasally (30 µL, 15 µL/nare) under the ketamine (80–100 mg/Kg) and xylazine (5–10 mg/Kg) anesthesia (21). DMEM-inoculated mice were used as uninfected controls for cytokine and chemokine analysis. After infection, body mass and rectal temperature were recorded daily for 21 dpi to determine disease severity. Subsets of mice were euthanized at 3, 10, and 21 dpi to collect lung tissue for virus titration, histopathology, flow cytometry, and cytokine and chemokine measurements. During the observation period, if any mouse lost ≥25% of its baseline body mass, had a body temperature below 32^0^C, or showed severe respiratory signs, it was humanely euthanized (21). For euthanasia, mice were anesthetized with a ketamine (80–100 mg/Kg) and xylazine (5–10 mg/Kg) cocktail or CO_2_ overdose (30 – 70% chamber volume displacement rate), followed by cervical dislocation, as per the approved protocol.

### 2.5 Virus titration

For virus titration, the MDCK cell-based 50% tissue culture infectious dose (TCID_50_) assay was performed (22). Briefly, 10-fold serially diluted lung homogenates were transferred to six replicates of MDCK cells in 96-well cell culture plates and incubated in a 32^0^C and 5% CO_2_ incubator. After 6 days of incubation, cells were fixed with 4% formaldehyde solution, stained with naphthol blue-black solution, and virus titers were determined using the Reed and Muench method.

### 2.6 Antibody measurement

In-house standardized enzyme-linked immunosorbent assays (ELISAs) were used to measure IgG, IgG1, and IgG2c antibodies in the plasma and IgG and IgA antibodies in lung homogenates (21, 23). Plasma and lung homogenates were two-fold serially diluted starting with 1:250 and 1:20 dilutions, respectively. Horseradish peroxidase (HRP)-conjugated secondary antibodies used were IgG (#31430, Invitrogen, MA, USA), IgG1 (#PA1-74421, Invitrogen, MA, USA), IgG2c (#56970, Cell Signaling Technologies, MA, USA), and IgA (#626720, Invitrogen, MA, USA) (21). Virus-neutralizing antibody titers were measured using the MDCK cell-based microneutralization assay (21).

### 2.7 Histopathology

Left lung lobes were inflated with aqueous buffered zinc formalin fixative (Z-fix, Anatech Ltd., MI, USA) on the day of necropsy and immersed in the fixative for a minimum of 72 hours before sending to the Kansas Veterinary Diagnostic Laboratory (KVDL) for histological processing. For comprehensive analysis, the left lung was sectioned into four quadrants, ensuring representative sampling across different anatomical regions. Fixed lung tissues were embedded in paraffin, sectioned at a thickness of 5 µm, mounted on glass slides, and stained with hematoxylin and eosin (H&E) for microscopic evaluation (19). A board-certified veterinary pathologist, blinded to the treatment groups, conducted a histopathological assessment. Inflammation of the pleura, parenchyma, vasculatures (arteries, veins, and capillaries), and airways (main bronchus, primary bronchi, and respiratory bronchioles) were each scored from 0 to 5. The cumulative lung inflammation score was between 0 to 40, reflecting the overall severity and extent of the inflammatory changes (20).

### 2.8 Cytokines/chemokines analysis

Cytokines and chemokines in the lung homogenates were measured using either the ProcartaPlex™ mouse cytokine and chemokine Panel 1, 26plex (#EPX260-26088-901) or mouse immune monitoring panel, 48plex (#EPX480-20834-901, Thermo Fisher Scientific, MA, USA) (21). The 26-plex included IL-1 family cytokines (i.e., IL-1β and IL-18); common gamma chain family cytokines (i.e., IL-2, IL-4, and IL-9); TNF-α; IL-10 family cytokines (i.e., IL-10 and IL-22); IL-12 family cytokines (i.e., IL-12p70, IL-23, and IL-27); IL-6, IL-17A, IL-5, and chemokines MCP-1, MIP-1α, GRO-α etc. Likewise, the 48-plex had interferons (IFN-α, IFN-γ, and IL-28 referred as IFN-λ); IL-1 family cytokines (i.e, IL-1α, IL-1β, IL-33, and IL-18); common gamma-chain cytokines (i.e., IL-2, IL-4, IL-7, IL-9, and IL-15); TNF superfamily cytokines (i.e., TNF-α, RANKL, and BAFF); IL-10 family cytokines (i.e, IL-10, IL-19, IL-22); IL-12 family cytokines (i.e., IL-12p70, IL-23, and IL27); IL-6 family cytokines (i.e., IL-6, IL-31, and LIF); different chemokines (i.e., MCP-1, MIP-1α, MIP-1β, RANTES, MCP-3, Eotaxin, GRO-α, MIP-2α, CXCL-5, and IP-10); various growth factors (i.e., BTC, G-CSF, GM-CSF, M-CSF, IL-3, and VEGF-A); and Th-2 cytokines IL-5 and IL-13. Plate reading was carried out using Luminex xMAP technology (TX, USA). For analysis, raw data were imported into the ProcartaPlex analysis application (Thermo Fisher Scientific, Waltham, MA, USA), and cytokine concentrations (pg/mL) were determined using a five-parameter line fitted to the standards provided by the manufacturer. For samples not having measurable cytokine concentration, half of the lowest detected value was used, while for concentrations beyond the highest detection limit, 1.5 times the highest detection limit was used to enable statistical comparisons (20, 21).

### 2.9 Preparation of cells for flow cytometry

Lungs were harvested, and single cells were prepared by enzymatic digestion (24). Briefly, right lung lobes were chopped into fine pieces, lung digestion medium containing DNAse type I (diluted to 10^4^ U/mL, 8µL/sample, #04536282001, Roche, Basel, Switzerland) and Gibco™ collagenase II (diluted to 10 mg/mL, 100 µL/sample, #17101015, Thermo Fisher Scientific, MA, USA) were added, and tubes were incubated (37 °C and 5% CO_2_ incubator) with intermittent shaking at 10 min intervals for 40 minutes. After incubation, lung pieces were pressed through the Fisherbrand™ 70 µm cell strainer (#22363548, Thermo Fisher Scientific, MA, USA) followed by lysis of red blood cells using Gibco™ ACK lysis buffer (#A1049201, Thermo Fisher Scientific, MA, USA) (24).

### 2.10 Flow cytometry

For staining with myeloid and lymphoid cell markers, 1 million cells were transferred into 96-well plates, Fc receptors were blocked using anti-CD16/32 antibody (#553141, BD Biosciences, CA, USA) and stained with panel-specific antibodies. Antibodies used in myeloid panels were BD Horizon V500 rat anti-mouse CD45 (0.2mg/mL, #561487, clone 30-F11), BD Horizon BV786 mouse anti-mouse CD64 (0.2mg/mL, #569507, clone X54-5/7.1), BD Horizon BV650 rat anti-CD11b (0.2mg/mL,#563402, clone M1/70), BD Pharmingen PE-Cy7 hamster anti-mouse CD11c (0.2mg/mL, #561022, clone HL3), BD Horizon RY610 rat anti-mouse Ly-6C (#571149, clone AL-21), BD Horizon BB700 rat anti-mouse Ly-6G (#566453, 1A8), BD Horizon BV421 rat anti-mouse CD86 (564198, GL1), BD Pharmingen PE rat anti-mouse Siglec-F (#562068, E50-2440), and BD Pharmingen Alexa Fluor 700 rat anti-mouse I-A/I-E (#570878, clone M5/114.15.2), BD Pharmingen Alexa Fluor 647 rat anti-mouse CD206 (#568808, clone Y17-505). Intracellular staining for CD206 was carried out following cell fixation with BD Cytofix fixation buffer (#554655) and permeabilization with BD Perm/Wash buffer (#554723).

Antibodies used in lymphoid panels were BD Horizon BV650 rat anti-mouse CD3 (#569683, clone 17A2), BD Pharmingen FITC rat anti-mouse CD4 (#561828, clone GK1.5), BD Horizon R718 rat anti-mouse CD8α (#566985, clone 53-6.7), BD Pharmingen PE-Cy7 rat anti-mouse CD19 (#561739, clone 1D3), BD Horizon BV421 mouse anti-mouse NK-1.1 (#562921, clone PK136), BD Horizon PE-CF594 hamster anti-mouse γδ T-cell receptor (#563532, clone GL3), BD Horizon RB705 rat anti-mouse CD25 (#570628, clone PC61), and BD Pharmingen PE rat anti-mouse Foxp3 (#563101, clone R16-715). For Foxp3 intracellular staining, cells were fixed and then permeabilized using BD Pharmingen Transcription Factor Buffer Set (#562574). Cells were acquired in LSR Fortessa X-20 (BD Biosciences, NJ, USA) and analyzed in FlowJo_v10.10.0_CL (BD Life Sciences, OR, USA).

From myeloid panel, frequencies of CD45^+^ cells, neutrophils (CD45^+^CD11b^+^Ly6G^+^), eosinophils (CD45^+^SiglecF^+^CD11c^-^), alveolar macrophages (CD45^+^SiglecF^+^CD11b^-/lo^CD11c^+^CD64^+^), interstitial macrophages (CD45^+^CD11b^+^MHC II^+^CD64^int/hi^), CD11b^+^ DC (CD45^+^CD11b^+^MHC II^+^CD64^-^), M1 macrophages (CD45^+^CD64^+^CD11b^+^CD86^+^), M2 macrophages (CD45^+^CD64^+^CD11b^+^CD206^+^), inflammatory (Ly6c^+^) monocytes/macrophages (CD45^+^CD64^+^CD11b^+^Ly6c^+^), and monocyte-derived dendritic cells (moDCs) (CD45^+^CD11b^+^MHCII^+/-^CD11c^+^Ly6c^+^) were determined (**Supplementary Figure 1**). Likewise, in lymphoid panels, frequencies of total T cells (CD3^+^), T helper cells (CD3^+^CD4^+^), CD8^+^ T cells (CD3^+^CD8^+^), regulatory T cells (CD3^+^CD4^+^CD25^+^Foxp3^+^), γδ T cells (CD3^+^γδ^+^), natural killer (NK) cells (CD3^-^NK1.1^+^), and B cells (CD3^-^B220^+^) were determined (**Supplementary Figure 2**).

### 2.11 Statistical analysis

Data were analyzed using GraphPad Prism version 10.5.0 and Microsoft Excel for Microsoft 365. Normality test was performed using the Shapiro-Wilk test. Normally distributed data were compared using an unpaired t-test or one-way ANOVA followed by Tukey’s *post-hoc* analysis, while non-normally distributed data were analyzed by the Mann-Whitney test or Kruskal-Wallis test followed by Dunn’s *post-hoc* comparisons for two or more than two groups, respectively. If repeated measurements were taken from the same animal, they were compared using repeated measures ANOVA (mixed effects model) with Tukey’s multiple comparisons. For cytokine and chemokine analysis, unpaired t-tests followed by Holm-Sidak correction for multiple comparisons were used for two groups, and one-way ANOVA or Kruskal-Wallis test followed by Benjamini and Hochberg multiple comparisons were used for more than two groups. Data were considered statistically significant at p<0.05 and having a trend at 0.05≤p ≤ 0.1.

## 3 Results

### 3.1 Males and females differed in metabolic biomarkers and in the progression of obesity

**Figure 1:**
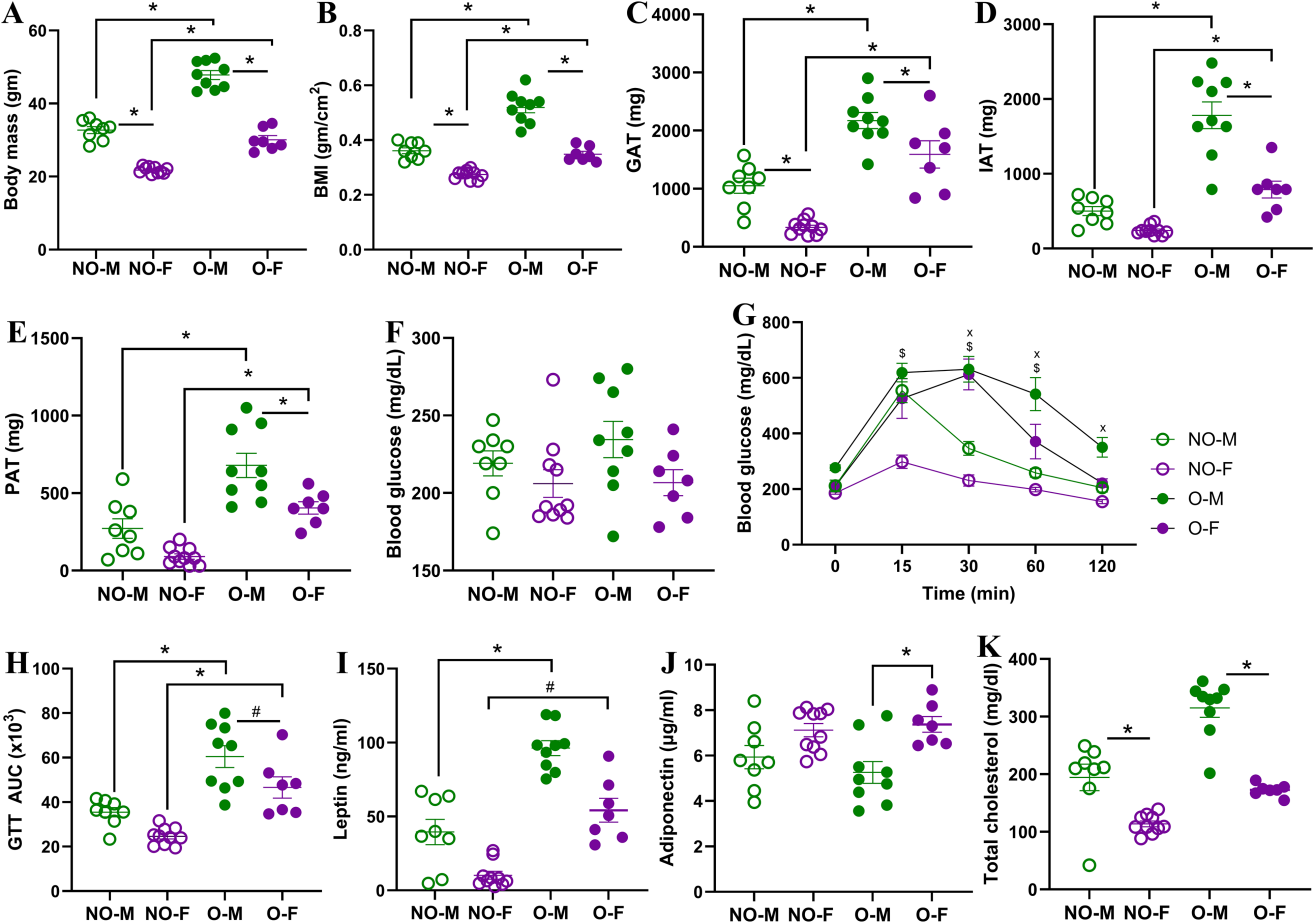
Progression of obesity and metabolic states in male and female mice. Male and female mice were fed with a low-fat diet (LFD) or a high-fat diet (HFD) for 14 weeks. Comparisons of **(A)** body mass after the 14^th^ week, **(B)** body mass index (BMI) on the day of infection, and **(C)** gonadal adipose tissue (GAT), **(D)** inguinal adipose tissue (IAT), and **(E)** perirenal adipose tissue (PAT) on day of euthanization (i.e., 3 dpi) are compared between male and female mice, with or without obesity. A glucose tolerance test (GTT) was performed on the 14^th^ week of diet treatment. Comparisons of **(F)** fed glucose, **(G)** GTT over time, and **(H)** GTT area under the curve (AUC) are shown. Comparisons of **(I)** leptin, **(J)** adiponectin, and **(K)** total cholesterol in plasma samples from blood collected at the 14^th^ week are shown. Data is presented as mean ± standard error of the mean (SEM) (n=7-10/group). Statistical comparisons were done using one-way ANOVA followed by Tukey’s *post-hoc* comparisons or Kruskal-Wallis test followed by Dunn’s *post-hoc* comparisons. Repeated measurements in 1G were compared by repeated measures ANOVA (mixed model) and Tukey’s multiple comparisons. An asterisk (*) indicates a statistically significant difference (p<0.05) while a hash (^#^) indicates a trend (0.05 ≤ p ≤ 0.1). In 1G, ‘x’ and ‘$’ represent significant differences between NO-M vs O-M, and NO-F vs O-F, respectively. NO-M, non-obese males; NO-F, non-obese females; O-M, males with obesity; and O-F, females with obesity.

Before the diet treatment (i.e., week 0), there was no difference in body mass between males on LFD (LFD-M) and HFD (HFD-M), or between females on LFD (LFD-F) and HFD (HFD-F). Males, however, had significantly greater body mass than the females (**Supplementary Figure 3A**). After the diet treatment, the percentage body mass change in male mice on HFD was significantly greater than that of their LFD controls within 1^st^ week, while it was substantially greater in females only from the 6^th^ week (**Supplementary Figure 3B**). While 100% of males on HFD met the obesity definition by gaining ≥20% body mass compared to the LFD-M within the 9^th^ week, only 70% of females on HFD were having obesity by the 14^th^ week, and 30% did not reach the obesity definition and were defined as non-responders (**Supplementary Figures 3C, D**). After 14 weeks, both male mice with obesity (O-M) and female mice with obesity (O-F) had significantly greater body mass, BMI, and deposition of gonadal, inguinal, and perirenal adipose tissues compared to non-obese males (NO-M) and non-obese females (NO-F), respectively (**Figures 1A–E**). Even after 14 weeks of diet treatment, males in each group were larger than the female mice. GTT on the 14^th^ week showed comparable fed glucose levels (**Figure 1F**). Male mice with obesity had significantly higher blood glucose levels at 30, 60, and 120 minutes, while female mice with obesity maintained such a difference at 15, 30, and 60 minutes. In addition, mice of both sexes with obesity had significantly higher GTT AUC values compared to their non-obese controls (**Figures 1G, H**).

At week 0, female mice had significantly higher plasma concentrations of leptin and adiponectin and significantly lower concentrations of total cholesterol than males (**Supplementary Figures 3E–G**). However, after the 14^th^ week of diet treatment, leptin concentration was significantly higher in males with obesity and showed a higher trend in females with obesity compared to their non-obese controls (**Figure 1I**). At 14 weeks, adiponectin concentration was significantly higher only in females with obesity compared to males with obesity (**Figure 1J**). Although mice with obesity had higher mean concentrations of total cholesterol at 14 weeks compared to their non-obese counterparts (NO-M= 194.5 mg/dL, O-M= 315.2 mg/dL, NO-F= 113.7 mg/dL, and O-F= 172.8 mg/dL), the differences were not statistically significant (**Figure 1K**). When we compared the fold increase in these biomarkers from week 0 to the 14^th^ week of diet treatment, males with obesity had an increased fold change of leptin (p=0.09) and significantly higher fold change in total cholesterol (p<0.05), while females with obesity had a higher trend of total cholesterol compared to their respective non-obese controls (**Supplementary Figures 3H–J**). Overall, these data indicate that the progression of obesity is quicker and more severe in males than in females on HFD, and males and females differ in terms of metabolic biomarkers before and after the diet treatments. Yet, the responder females (i.e., those with ≥20% body mass gain) showed significantly increased body mass, BMI, adipose tissue deposition, glucose intolerance, and higher leptin and total cholesterol levels than the non-obese females, as observed between males, confirming obesity status.

### 3.2 After a high-dose influenza virus infection, the median survival time was shorter for females with or without obesity than for respective males

**Figure 2:**
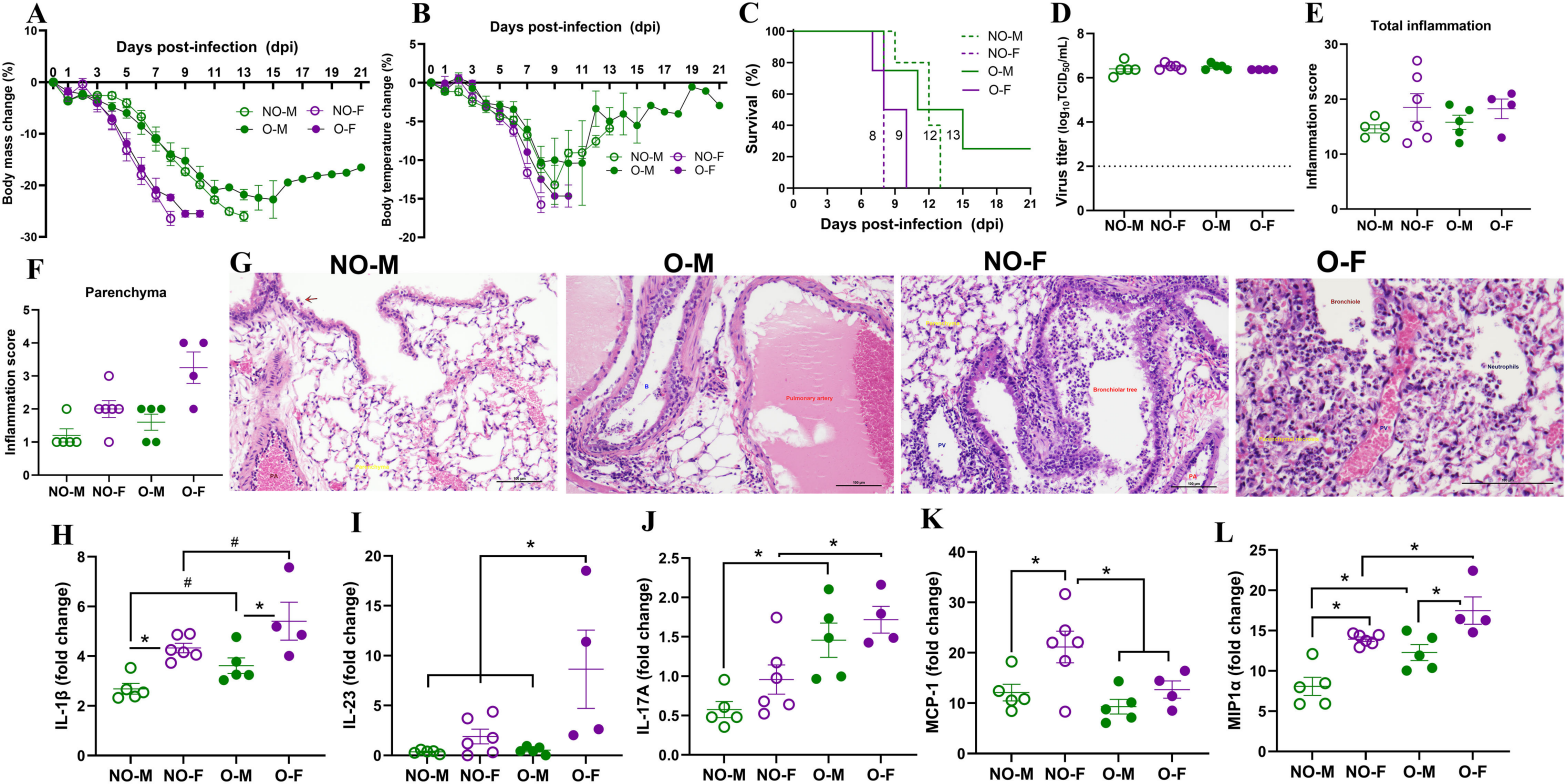
Morbidity, virus replication, lung histopathology, and cytokine and chemokine responses following a high-dose IAV infection. Male and female mice, with or without obesity, were intranasally inoculated with a high dose (10^3^ TCID_50_) of the 2009 pandemic H1N1 IAV or vehicle (i.e., medium) only and monitored for body mass and body temperature changes. **(A)** Percentage change in body mass and **(B)** body temperature, and **(C)** survival percentages are shown (n=4-5/group). Subsets of mice (n=3-6/group) were euthanized at 3 days post-infection (dpi); **(D)** lung viral titers were measured by TCID_50_ (dashed line, limit of detection), and **(E–G)** histopathology analysis was performed in the lungs by H & E staining. **(E)** Total inflammation scores and **(F)** parenchymal inflammation between the four virus-infected groups are presented. **(G)** Representative histopathology images of non-obese males (NO-M), males with obesity (O-M), non-obese females (NO-F), and females with obesity (O–F) are shown. Likewise, (H–L) fold changes in cytokines and chemokines IL-1β, IL-23, IL-17A, MCP-1, and MIP-1α in virus-infected mice compared to their medium-inoculated controls were compared. Data is presented as mean ± standard error of the mean (SEM). Statistical comparisons were done using one-way ANOVA followed by Tukey’s *post-hoc* comparisons or Kruskal-Wallis test followed by Dunn’s *post-hoc* comparisons. For cytokine analysis, p-values were adjusted for multiple comparisons using the Benjamini-Hochberg method. An asterisk (*) indicates a statistically significant difference (p<0.05) while a hash (^#^) indicates a trend (0.05 ≤ p ≤ 0.1).

After infection with a high dose (i.e. 10^3^ TCID_50_) of the 2009 pandemic H1N1 IAV, all virus-infected mice showed gradual body mass loss and reached the humane endpoint (i.e., ≥25%) except for one male with obesity which lost up to 21% of its body mass and did not recover even at 21 dpi when it still maintained 17% body mass loss (**Figure 2A**). Likewise, body temperature decreased in infected mice until 10 dpi or when they reached their humane endpoint (**Figure 2B**). Sex difference in survival was observed in mice both with or without obesity. Among non-obese mice, females showed a significantly shorter median survival time (8 days) compared to non-obese males (12 days; p<0.05). Likewise, females with obesity had a trend toward a shorter median survival time (9 days) compared to the males with obesity (13 days; p= 0.07) (**Figure 2C**). At 3 dpi, between 6 and 7 log_10_ TCID_50_ of infectious virus titer was determined in the lungs of virus-infected mice. However, there was no statistical difference in infectious virus titers in the lungs by biological sex or obesity status of the mice (**Figure 2D**), indicating that sex difference in disease severity was not attributed solely to differences in pulmonary virus replication.

To test if the severe disease in females is associated with greater pulmonary inflammation, we performed histopathology analysis of H&E-stained lungs at 3 dpi. Inflammation in the pleura, lung airways, vessels, and parenchyma was evaluated. While there were no obvious lesions in lungs from medium-inoculated mice, bronchopneumonia and arteritis were evident in IAV-infected male and female mouse lungs, with or without obesity (**Supplementary Figures 4A, B**). Among virus-infected mice, there was no significant difference in total lung inflammation score (**Figure 2E**). Likewise, inflammatory responses were comparable among the four groups in the pleura, lung airways, and pulmonary vessels (**Supplementary Figures 4C–E**). Interestingly, although the differences were not statistically significant, lung parenchymal inflammation was more severe in female mice with obesity compared to all other groups, as indicated by average inflammation scores in the parenchyma (i.e., 1.2 for NO-M, 2 for NO-F, 1.6 for O-M, and 3.3 for O-F, out of the maximum score of 5) (**Figures 2F, G**). These data suggest that following high-dose IAV infection, female mice, with or without obesity, exhibit shorter survival time than males, which is not driven by lung virus titers. While the overall pulmonary inflammation is comparable, females with obesity display more severe parenchymal inflammation than other groups.

### 3.3 A high-dose IAV infection induced a strong pulmonary cytokine and chemokine response in all groups

To further compare inflammatory changes, we measured 26 cytokines and chemokines in the lung homogenates at 3 dpi. Infection with high-dose IAV induced a strong inflammatory response in the lungs of mice from all groups. It was characterized by significantly elevated absolute concentrations of pro-inflammatory cytokines (IL-6, TNF-α, IL-1β, and IL-18), antiviral mediator (IFN-γ), chemokines involved in neutrophil and monocyte recruitment (GRO-α, MIP-2α, MCP-1, MCP-3, MIP-1α, and MIP-1β), myeloid cell activation cytokine such as GM-CSF, and interferon-inducible chemokine IP-10, irrespective of biological sex and obesity (**Supplementary Figures 5A–D**). However, concentrations of the T helper type 1 (Th1)-polarizing cytokines IL-12p70 and IL-27, and lymphocyte-recruiting chemokine RANTES showed a higher trend or significant difference in virus-infected mice compared to medium-inoculated mice from three groups, except for non-obese males (**Supplementary Figures 5A–D**). Likewise, IL-22, involved in tissue repair and epithelial regeneration, showed a higher trend only in virus-infected males and females with obesity, but not in non-obese mice. When the concentrations of cytokines and chemokines were compared among virus-infected groups, a statistical difference was observed only for the T helper type 2 (Th2)-associated mediators eotaxin and IL-5, which were both lower in virus-infected non-obese females compared to non-obese males and females with obesity (**Supplementary Figure 5E**).

To determine the magnitude of changes in pulmonary cytokines and chemokines across the groups, we determined fold changes in virus-infected mice relative to their respective medium-inoculated controls. The relative induction of proinflammatory cytokine IL-1β was significantly higher in females than in males, irrespective of obesity status (**Figure 2H**). Among the T helper type 17 (Th-17)-associated cytokines, fold change of IL-23 was significantly greater in females with obesity than in all other groups, whereas IL-17A fold change was significantly greater in both males and females with obesity (**Figures 2I, J**). Among the leucocyte recruitment chemokines, fold change for MCP-1α was significantly greater in non-obese females compared to other groups, while MIP-1α fold change was greatest in females with obesity (**Figures 2K, L**). These data indicate that a high-dose IAV infection induces a strong inflammatory response in all groups, with unique sex- and obesity-specific alterations in certain pulmonary cytokines and chemokines.

### 3.4 After a low-dose influenza virus infection, females with obesity suffered from more severe disease, which was not associated with lung virus titers

**Figure 3:**
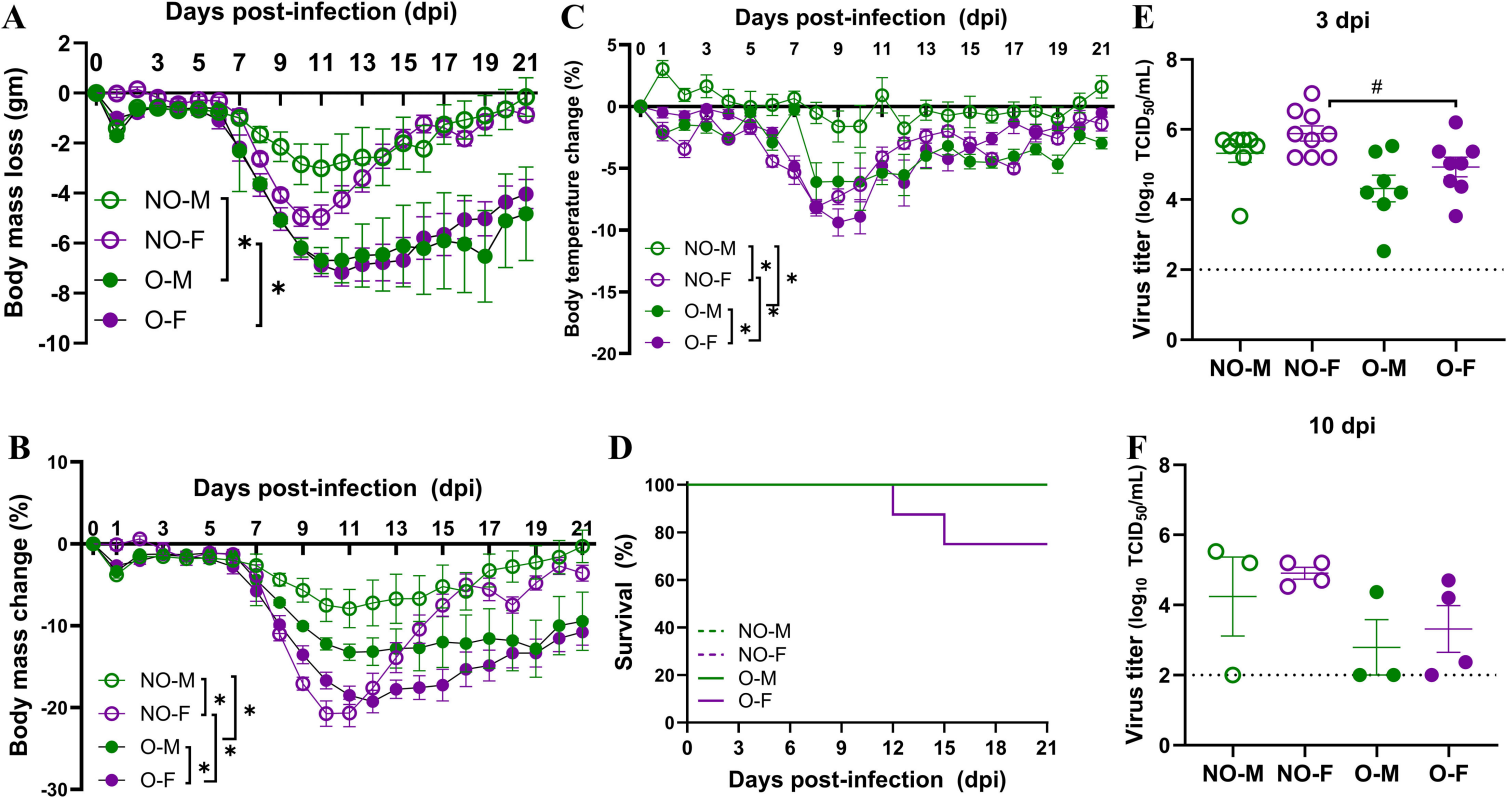
Morbidity and virus replication following a low-dose IAV infection. Male and female mice, with or without obesity, were intranasally inoculated with a low dose (i.e., 10^1.5^ TCID_50_) of the 2009 pandemic H1N1 IAV and monitored for 21 days post-infection (dpi) for changes in body mass and temperature. **(A)** Absolute (gm) and **(B)** relative (%) changes in body mass and **(C)** relative (%) change in body temperature, and **(D)** survival curves are compared (n=5-8/group). Virus titers in the lungs were measured by TCID_50_ assay at **(E)** 3 dpi (n=7-9/group) and **(F)** 10 dpi (n=3-4/group). Data is shown as mean ± standard error of the mean (SEM). Statistical comparisons were done using one-way ANOVA followed by Tukey’s *post-hoc* comparisons or Kruskal-Wallis test followed by Dunn’s *post-hoc* comparisons. Repeated measurements in A-C were compared by repeated measures ANOVA (mixed model) and Tukey’s multiple comparisons. An asterisk (*) indicates a statistically significant difference (p<0.05) while a hash (^#^) indicates a trend (0.05 ≤ p ≤ 0.1). NO-M, non-obese males; NO-F, non-obese females; O-M, males with obesity; and O-F, females with obesity.

After infection with a low dose of IAV (i.e., 10^1.5^ TCID_50_), the absolute (from 10–21 dpi) and relative (on 15, 17 to 21 dpi) body mass losses were significantly higher in males with obesity compared to non-obese males (**Figures 3A, B**). A similar effect of obesity was observed in females, where the absolute (from 13–21 dpi) and relative (from 14–21 dpi) body mass losses were significantly higher in females with obesity compared to non-obese females (**Figures 3A, B**). We also compared the biological sex differences in body mass loss. The percentage change in body mass was significantly greater in non-obese females from 9–13 dpi compared to non-obese males, while it was significantly greater in females with obesity compared to males with obesity between 14–15 dpi (**Figure 3B**). Though peak body mass loss occurred between 9–13 dpi, and females, regardless of obesity status, experienced the greatest losses, non-obese mice recovered to their baseline body mass by 21 dpi, whereas mice with obesity did not (**Figures 3A, B**). The percentage change in body temperature also showed a similar trend, where mice with obesity, irrespective of sex, lost significantly greater body temperature than the non-obese mice; and females, with or without obesity, lost significantly greater temperature, indicating more severe disease (**Figure 3C**).

Morbidity was most severe in females with obesity, which is evident from the observation that even at a low-dose infection, 2 of 8 (i.e., 25%) females with obesity lost ≥25% of their original body mass and reached humane endpoints, while all mice from other groups survived (**Figure 3D**). To determine if the difference in viral replication in the lungs contributes to the differences in disease severity among different groups, we euthanized subsets of mice at 3 and 10 dpi and collected lung tissues for virus titration. At 3 dpi, there was a lower trend (p= 0.1) of virus titers in the lungs of females with obesity as compared to the non-obese females (**Figure 3E**). Virus was detected in the lungs of mice even at 10 dpi, with no statistical difference observed (**Figure 3F**). These data suggest that obesity is associated with increased disease severity following IAV infection in both males and females. Furthermore, sex difference is observed in disease severity following the low-dose IAV infection in mice with obesity, and females suffer from more severe disease than males.

### 3.5 Female mice with obesity had delayed but persistent histopathological changes after a low-dose IAV infection up to 21 dpi

**Figure 4:**
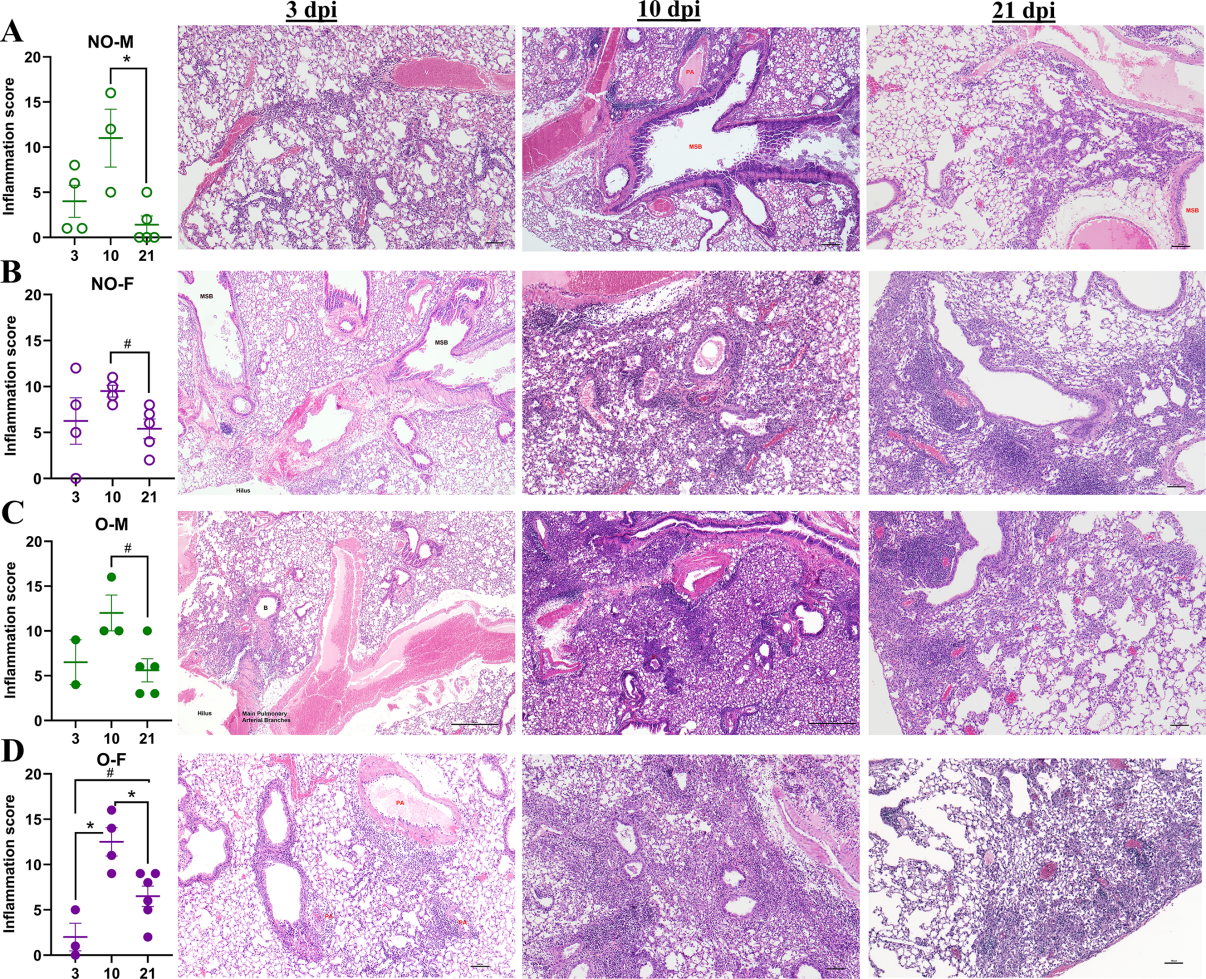
Progression of lung inflammation following low-dose IAV infection. Male and female mice, with or without obesity, were intranasally inoculated with a low dose (i.e., 10^1.5^ TCID_50_) of the 2009 pandemic H1N1 IAV. Subsets of mice were euthanized at 3, 10, and 21 dpi, and histopathology analysis by hematoxylin and eosin (H&E) staining was performed. Total inflammation scores at 3, 10, and 21 dpi together with their representative images for the **(A)** non-obese males (NO-M), **(B)** non-obese females (NO-F), **(C)** males with obesity (O–M), and **(D)** females with obesity (O–F) are shown (n=2-6/group). Data is shown as mean ± standard error of the mean (SEM). Statistical comparisons were performed using the one-way ANOVA or Kruskal-Wallis test followed by Tukey’s or Dunn’s multiple comparisons, respectively. An asterisk (*) indicates a statistically significant difference (p<0.05) while a hash (^#^) indicates a trend (0.05 ≤ p ≤ 0.1). V, vessel; PA, pulmonary artery; MSB, main-stem bronchi; Br, bronchiole.

Following a low-dose IAV infection, mice were euthanized at 3, 10, and 21 dpi, and pulmonary pathological changes across these timepoints were compared (**Figure 4**). In non-obese males, inflammation was observed at 3 dpi (average score: 4), peak inflammation was at 10 dpi (average score: 11), and there was a significant decline in inflammation by 21 dpi (average score: 1.4) (**Figure 4A**). Representative images of non-obese males at 3 dpi showing inflammation mainly centered on vessels (V); 10 dpi showing inflammation centered on airways and arteries; and 21 dpi showing residual parenchymal inflammation around the main stem bronchus (MSB) are shown (**Figure 4A**). In non-obese females, inflammation was relatively stronger at 3 dpi (average score: 6.3); reached a peak at 10 dpi (average score: 9.5); and there was a lower trend by 21 dpi (average score: 5.4) (**Figure 4B**). Representative images of non-obese females at 3 and 10 dpi, showing multifocal inflammation, and at 21 dpi with residual inflammation are shown (**Figure 4B**). In males with obesity, inflammation was relatively stronger at 3 dpi (average score: 6.5), peaked at 10 dpi (average score: 12), and there was a lower trend by 21 dpi (average score: 5.6) (**Figure 4C**). Representative images of males with obesity at 3, 10, and 21 dpi are shown (**Figure 4C**). Interestingly, in females with obesity, inflammation at 3 dpi was relatively weaker (average score: 2) significantly increased at 10 dpi (average score: 12.5); and there was a significant reduction in inflammation at 21 dpi compared to 10 dpi, but inflammation at 21 dpi still had a higher trend than at 3 dpi (average score: 6.5) (**Figure 4D**). Representative images for females with obesity at 3, 10, and 21 dpi are shown (**Figure 4D**). Overall, these data suggest that groups other than non-obese males had residual inflammation even at 21 dpi. Females with obesity had a delayed onset of pulmonary pathological changes that increased significantly at 10 dpi and persisted longer up to 21 dpi, resembling severe morbidity and delayed recovery.

### 3.6 Female mice with obesity showed delayed but persistent pulmonary cytokine and chemokine influx following a low-dose IAV infection

**Figure 5:**
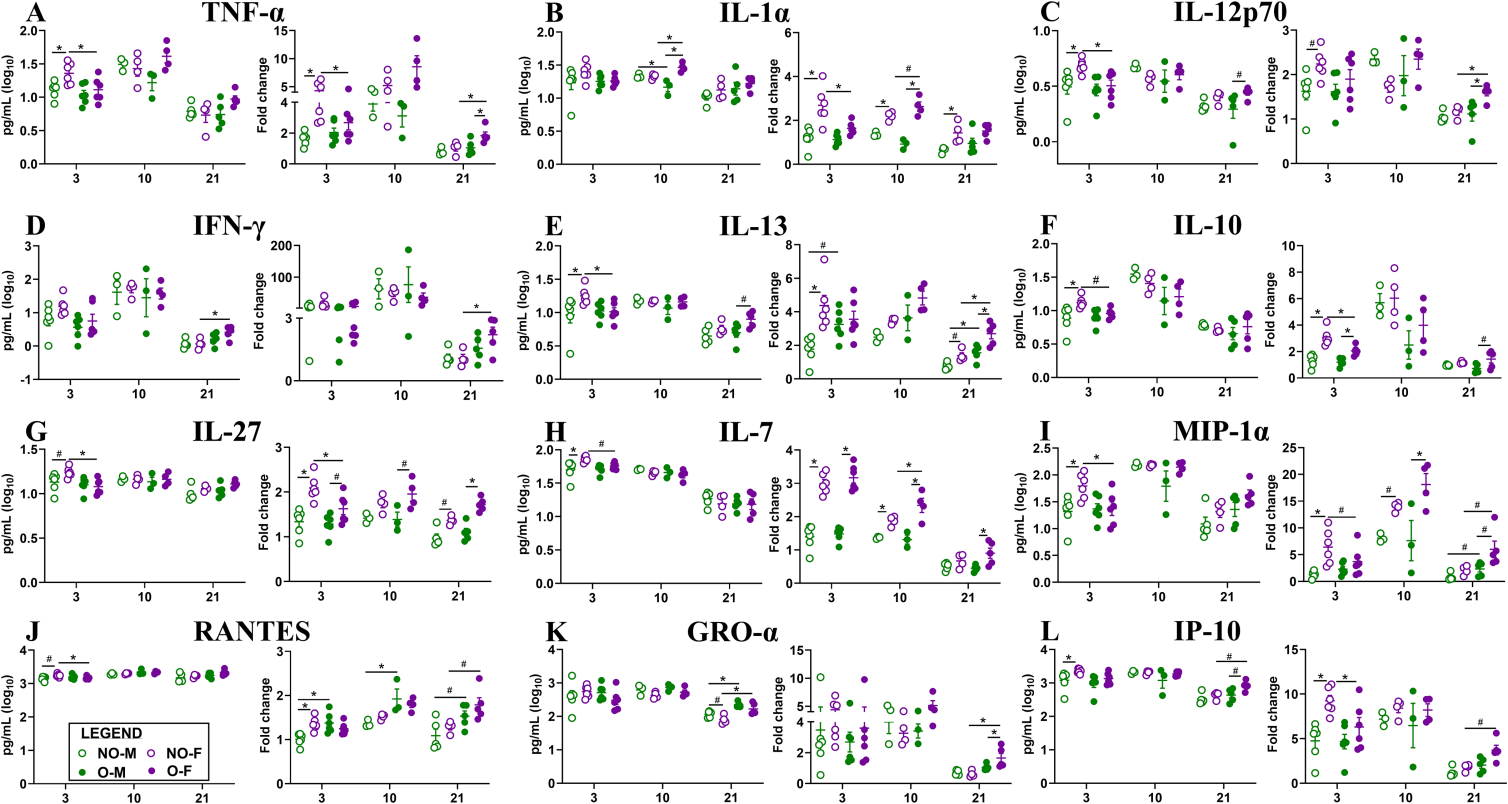
Cytokine/chemokine responses in the lungs following low-dose IAV infection. After infection with a low dose (i.e., 10^1.5^ TCID_50_) of the 2009 pandemic H1N1 IAV, subsets of mice were euthanized at 3-, 10-, and 21-days post-infection (dpi), lungs were collected, and various cytokines and chemokines were measured. Fold changes in cytokines and chemokines in virus-infected mice at different days were determined by comparing to their respective medium-inoculated controls euthanized at 3 dpi. Absolute concentrations and fold changes for **(A)** TNF-α, **(B)** IL-1α, **(C)** IL-12p70, **(D)** IFN-γ, **(E)** IL-13, **(F)** IL-10, **(G)** IL-27, **(H)** IL-7, **(I)** MIP-1α, **(J)** RANTES, **(K)** GRO-α, and **(L)** IP-10, for 3-, 10-, and 21-dpi are shown. Statistical comparisons were made using one-way ANOVA or Kruskal-Wallis test, and p-values were adjusted for multiple comparisons using the Benjamini-Hochberg method. An asterisk (*) indicates a statistically significant difference (p<0.05) while a hash (^#^) indicates a trend (0.05 ≤ p ≤ 0.1). NO-M, non-obese males; NO-F, non-obese females; O-M, males with obesity; and O-F, females with obesity.

After the low-dose virus infection, we measured 48 cytokines and chemokines in the lung homogenates at 3 dpi, 10 dpi, and 21 dpi. At 3 dpi, the absolute concentrations of cytokines and chemokines were greater in non-obese females compared to the other groups (**Figure 5**; **Supplementary Figure 6**). Specifically, non-obese females had significantly higher concentrations of proinflammatory cytokines TNF-α and IL-18; Th1-associated cytokine IL-12p70; T cell survival and homeostasis cytokines IL-2R and IL-7Rα; Th2-associated cytokines IL-4, IL-9, and IL-13; regulatory cytokine IL-27; tissue repair and epithelial regeneration cytokine IL-22; epidermal growth factor BTC; chemokines involved in myeloid cell differentiation and monocyte recruitment MCP-3 and MIP-1α; myeloid cell activation cytokine GM-CSF; and T cell recruitment chemokine RANTES compared to females with obesity (**Figure 5**; **Supplementary Figure 6**). Likewise, they had a higher trend for the concentrations of antiviral cytokine IL-28; T cell survival and homeostasis cytokines IL-2, IL-7, and IL-15; anti-inflammatory and regulatory cytokines IL-10 and LIF; Th17-associated cytokine IL-17A; and macrophage recruitment chemokine MIP-1β compared to females with obesity at 3 dpi (**Figure 5**; **Supplementary Figure 6**). At 10 dpi, the concentrations of cytokines and chemokines were mostly comparable among the groups. However, the concentration of the proinflammatory cytokine IL-1α was significantly higher in females with obesity than in non-obese females (**Figure 5B**). At 21 dpi, females with obesity had significantly higher concentrations of antiviral cytokine IFN-γ; chemokines for neutrophil recruitment GRO-α; and chemokine for monocyte recruitment MCP-1; and a higher trend for Th1-associated chemokine IP-10 and Th2-associated cytokine IL-33 compared to the non-obese females (**Figure 5**; **Supplementary Figure 6**).

A comparison of the magnitude of cytokine and chemokine induction by fold changes further ascertained these differences. At 3 dpi, non-obese females showed significantly higher fold changes or induction of proinflammatory cytokines IL-1α, TNF-α, and IL-18; antiviral and Th1-associated cytokines IL-28, IP-10, and IL-27; T-cell survival and homeostasis cytokines IL-2 and IL-2R; Th2-associated cytokines IL-4; anti-inflammatory and regulatory cytokine IL-10 and LIF; chemokines involved in myeloid cell differentiation and monocyte recruitment MCP-1, and MCP-3; myeloid cell activation cytokines GM-CSF and M-CSF; osteoclast activation and immune signaling cytokine RANKL; and lymphocyte recruiting chemokine MIP-1β than females with obesity (**Figure 5**; **Supplementary Figure 7**). They also had a higher trend in fold changes for MIP-1α, eotaxin, G-CSF, and BAFF at 3 dpi. At 10 dpi, females with obesity had significantly higher fold changes in IL-7, IL-9, and a higher trend for IL-1α and IL-18 compared to non-obese females. At 21 dpi, females with obesity had significantly higher fold changes for TNF-α, IFN-γ, IL-13, GRO-α, and a higher trend for IFN-α, IL-15, MIP-1α, RANTES, IP-10, IL-3, and BTC than non-obese females (**Figure 5**; **Supplementary Figure 7**). The fold change for eotaxin, however, was significantly lower in females with obesity than in non-obese females at 21 dpi (**Supplementary Figure 7**). These data suggest that in non-obese females, cytokines and chemokines were induced at high levels at 3 dpi, which was maintained up to 10 dpi, and declined by 21 dpi; while in females with obesity, the levels were lower at 3 dpi, reached comparable levels to non-obese females at 10 dpi, but remained higher even at 21 dpi. The cytokine and chemokine data corroborate with the histopathology data, indicating delayed but persistent inflammation in females with obesity.

### 3.7 Pulmonary myeloid and lymphoid cell frequencies during the acute phase were lower in females with obesity

**Figure 6:**
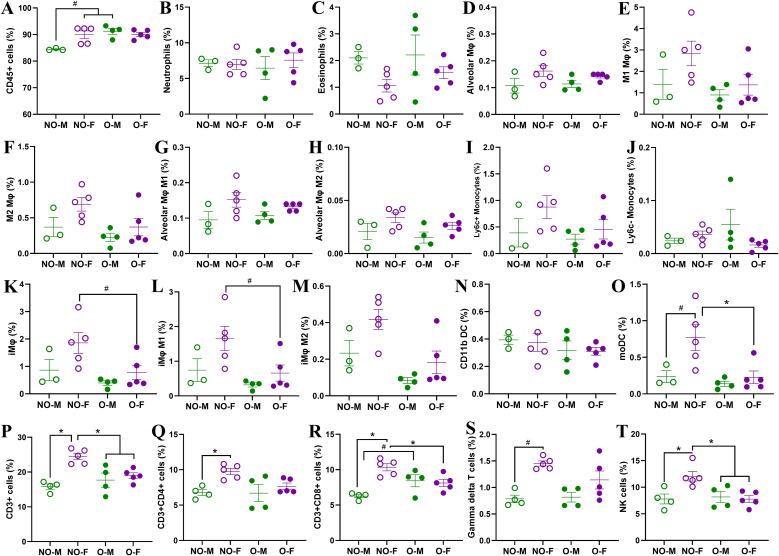
Characterization of myeloid and lymphoid cells in the lungs following a low-dose IAV infection. After infection with a low dose (i.e., 10^1.5^ TCID_50_) of the 2009 pandemic H1N1 IAV, subsets of mice were euthanized at 3 days post-infection (dpi) to determine the frequencies of various myeloid and lymphoid cells in the lungs. In myeloid panel, the frequencies of **(A)** CD45^+^ cells, **(B)** neutrophils, **(C)** eosinophils, **(D)** alveolar macrophages, **(E)** M1 macrophages, **(F)** M2 macrophages, **(G)** M1 and **(H)** M2 type alveolar macrophages, **(I)** Ly6c^+^ and **(J)** Ly6c^-^ monocytes, **(K)** interstitial macrophages, **(L)** M1 and **(M)** M2 type interstitial macrophages, **(N)** CD11b dendritic cells, and **(O)** monocyte-derived dendritic cells are compared. Likewise, frequencies of **(P)** CD3^+^ T cells, **(Q)** CD3^+^CD4^+^ T helper cells, **(R)** CD3^+^CD8^+^ T cells, **(S)** gamma delta T cells, and **(T)** NK cells are compared. Data is shown as mean ± standard error of the mean (SEM) (n=3-5/group). Statistical comparisons were performed using the one-way ANOVA or Kruskal-Wallis test, followed by Tukey’s or Dunn’s multiple comparisons, respectively. An asterisk (*) indicates a statistically significant difference (p<0.05) while a hash (^#^) indicates a trend (0.05 ≤ p ≤ 0.1). NO-M, non-obese males; NO-F, non-obese females; O-M, males with obesity; and O-F, females with obesity.

At 3 dpi, frequencies of myeloid and lymphoid cells were determined in the lungs. CD45^+^ cells had a lower trend in non-obese males compared to non-obese females and males with obesity, and there was no difference in the frequencies of neutrophils and eosinophils (**Figures 6A–C**). The frequencies of alveolar macrophages, M1 and M2 macrophages, and Ly6c^+^ inflammatory monocytes were all higher in non-obese females compared to other groups but did not reach statistical significance (**Figures 6D–J**). The interstitial macrophages, including the M1 types, showed a higher trend in non-obese females than in females with obesity (**Figures 6K–M**). There was no difference in CD11b DC frequencies, while the frequencies of moDCs were significantly higher in non-obese females compared to the females with obesity and showed a higher trend than in non-obese males (**Figures 6N,O**).

Among the lymphoid cells, the frequencies of CD3^+^ cells were significantly higher in non-obese females compared to the other groups (**Figure 6P**). The frequency of T helper (CD3^+^CD4^+^) cells was also significantly higher in non-obese females compared to non-obese males (**Figure 6Q**). The CD3^+^CD8^+^ T cells were significantly higher in non-obese females compared to non-obese males and females with obesity. Males with obesity also had a higher trend of CD3^+^CD8^+^ T cells compared to non-obese males (**Figure 6R**). There was no difference in the frequencies of regulatory T cells at 3 dpi in the lungs. However, the gamma delta T cells were highest in the non-obese females, which showed a higher trend compared to non-obese males. Likewise, the NK cell frequency was significantly higher in non-obese females compared to all other groups (**Figures 6S, T**). Together, these data suggest that deposition of myeloid and lymphoid cells at 3 dpi was also greater in non-obese females compared to the other groups, including the females with obesity.

### 3.8 Female mice, with or without obesity, had higher antibody induction compared to the male mice following a low-dose IAV infection

At 21 dpi, antibody responses were compared in plasma (**Supplementary Figures 8A–D**) and lung homogenates (**Supplementary Figures 8F–H**). In plasma, IgG and IgG2c antibodies were significantly higher in non-obese females compared to non-obese males, and females with obesity had significantly higher IgG and a higher trend of neutralizing antibodies (nAb) than males with obesity (**Supplementary Figures 8A–D**). In the lungs, total B-cell frequency was determined by flow cytometry at 3 dpi, and non-obese female mice had significantly lower frequency than non-obese males and females with obesity (**Supplementary Figure 8E**). Non-obese females had significantly higher IgG and a higher but not significant increase in IgA and nAb titers in the lungs compared to non-obese males (**Supplementary Figures 8F–H**). Likewise, among mice with obesity, antibody production was higher among females than in males. For example, while 100% of females with obesity exhibited IgA and nAb titers above the detection limit, only 40% of males with obesity did so. However, these data were not statistically significant (**Supplementary Figure 8H**). Overall, these data indicate that females, irrespective of obesity, have stronger antibody induction locally and systemically compared to their male counterparts at 21 days following infection with a low-dose IAV.

### 3.9 Non-responder females on HFD had morbidity, virus titers, and pulmonary inflammation comparable to females with obesity

**Figure 7:**
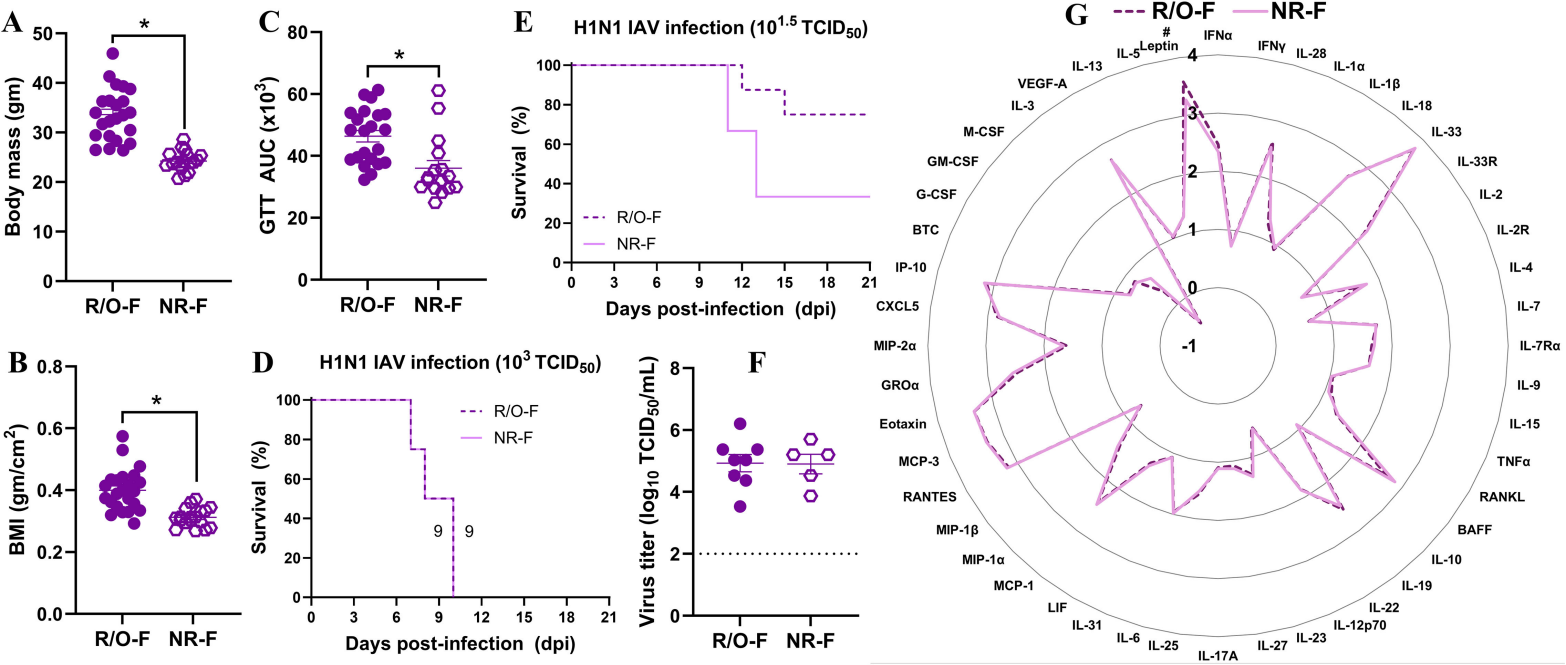
Comparison of morbidity, virus replication, and pulmonary cytokine and chemokine responses between HFD-treated responder and non-responder females. Female mice were on HFD for 13 to 14 weeks. About 30% of them were non-responders. **(A)** Body mass, **(B)** body mass index (BMI), and **(C)** glucose tolerance test (GTT) area under the curve (AUC) between females with obesity (i.e., the responders) and non-responder females are compared (n=16-23/group). Likewise, **(D)** survival rates after high-dose IAV infection and **(E)** low-dose IAV infection are shown (n=4-8/group). Similarly, **(F)** virus titers and **(G)** log_10_-transformed cytokine and chemokine concentrations in the lungs at 3 dpi with a low-dose virus infection are compared (n=5-8/group). Data is shown as mean ± standard error of the mean (SEM). Statistical comparisons were made using the unpaired t-test or Mann-Whitney test, and an asterisk (*) indicates a statistically significant difference (p<0.05) while a hash (^#^) indicates a trend (0.05 ≤ p ≤ 0.1).

As some of the females on HFD were non-responders, we compared influenza virus infection outcomes between non-responder females and responder (i.e., having obesity) females. The body mass, BMI, and GTT values were significantly lower in non-responder females compared to the females with obesity (**Figures 7A–C**). After a high-dose infection, the median survival time was 9 dpi for both groups (**Figure 7D**). After a low-dose infection, 20% of the obese females and 80% of the non-responders were euthanized as they reached the humane endpoint (p=0.1, **Figure 7E**). We also compared virus replication in the lungs after infection with the low dose at 3 dpi, and it was similar between the groups (**Figure 7F**). To determine if the induction of inflammatory cytokines and chemokines differs between the non-responder and responder groups, we also compared the 48 cytokines/chemokines at 3 dpi, and there were similar responses (**Figure 7G**). The only difference observed was in leptin concentration, which showed a higher trend in the females with obesity than in non-responder females (**Figure 7F**). These data suggest that, despite not gaining sufficient body mass to be defined as having obesity in this model, prolonged intake of HFD in non-responder females increased the risk of severe IAV infection outcomes, comparable to those observed in females that developed obesity.

## 4 Discussion

In this study, we aimed to determine sex differences in influenza pathogenesis and disease severity during obesity using a DIO mouse model and both high- and low-dose infection models of the 2009 pandemic H1N1 IAV. Our findings suggest that sex difference is observed following IAV infection during obesity. Females with obesity suffer from more severe disease compared to males, and it is likely associated with the delayed but persistent pathological changes in the lungs.

After the HFD treatment, male mice developed obesity earlier than females. This is consistent with prior findings suggesting that female mice are more resistant to developing DIO compared to males (19, 25). Such resistance in females is likely associated with greater energy expenditure, estrogen-mediated protection, and compensatory increase in genes related to appetite control and energy balance in the hypothalamus (25, 26). Female mice had higher baseline and 14th-week adiponectin concentrations than males. This might also contribute to slower body weight gain in females, as the adiponectin-to-leptin ratio is negatively correlated with body weight gain, adipose tissue deposition, and insulin resistance (27). In this study, we defined obesity as having a ≥20% body mass gain compared to the average body mass of age- and sex-matched mice on LFD treatment. Using this approach, around 70% of the females on HFD gained sufficient body mass to define them as having obesity. Females with obesity had greater body mass, BMI, adipose tissue deposition, glucose intolerance, and leptin concentrations compared to non-obese females; the observations were similar between males with or without obesity, making this approach a relevant model to study sex differences in IAV pathogenesis during obesity in both sexes.

Among non-obese mice, females exhibited greater influenza virus-driven disease severity. During the high-dose infection, female mice had a shorter median survival time (i.e., 8 vs 12 days) and a higher fold change induction of pro-inflammatory cytokine such as IL-1β and leucocyte recruiting chemokines such as MCP-1 and MIP-1α, compared with males at 3 dpi. Likewise, after a low-dose IAV infection, non-obese females exhibited significantly greater body mass loss and reduced body temperature, indicating greater disease severity than males. These findings are similar to earlier studies, which have shown that morbidity and mortality from H1N1 influenza virus infection are greater in lean females than in lean males (16, 17, 21, 28). Non-obese females also had higher concentrations and fold changes of several inflammatory cytokines and chemokines at 3 dpi, including pro-inflammatory cytokines TNF-α and IL-18,; chemokines involved in monocytes, macrophages, and T cells recruitment such as MCP-1, MIP-1α, MIP-1β, and IP-10; and myelopoietic growth factors G-CSF and GM-CSF that drive myeloid cell proliferation and differentiation. This was also supported by the relatively higher deposition of myeloid and lymphoid cells in the lung tissues of non-obese females compared to non-obese males. The lung virus titers after both the high- and low-dose infection, however, were comparable between the non-obese males and females, indicating that observed sex differences in morbidity and mortality are not associated entirely with higher lung virus replication. Prior studies also suggest that sex differences during IAV infection are dose-dependent, and although lung virus titers are comparable between males and females, the higher disease severity observed in non-obese females is associated with heightened inflammatory responses and pulmonary tissue damage (17, 29). Higher testosterone levels and amphiregulin, involved in lung tissue repair, are also associated with better influenza outcomes in non-obese males compared to non-obese females (17).

Although studies investigating sex differences during influenza and other viral infections among hosts with obesity are limited, Lee et al. observed that following infection with the alpha variant of SARS-CoV-2 in the K18-hACE2 mouse model of DIO, female mice with obesity exhibited more severe symptoms and higher inflammatory lung burdens than males (30). Prior studies of influenza pathogenesis in mouse models of DIO used either male-only, female-only, or males and females, but without stratification of data by sex (18). One study examined both male and female DIO mice infected with a lethal dose of the virus and followed only up to 5 dpi, where they observed similar pathological changes after IAV infection in males and females with obesity (31). In our study, we investigated sex differences in influenza pathogenesis during obesity using both high- and low-dose infection models and following mice up to 21 dpi. After infection with the high-dose IAV, females with obesity had a shorter median survival time (9 versus 13 days) than males with obesity. Despite having comparable lung virus titers and total pulmonary inflammation, parenchymal inflammation was more severe in females than in males with obesity. Female mice also exhibited a stronger fold change in proinflammatory cytokine IL-1β and chemokine MIP-1α than males with obesity, suggesting greater activation of inflammatory mediators. Females with obesity also had greater fold increase in IL-23 and IL-17A cytokines, compared to males with obesity. This indicates a probable role of IL-23/IL-17 inflammatory pathway in mediating severe influenza disease in females with obesity, warranting further exploration by direct mechanistic studies involving blockade by antibodies or genetic ablation (32, 33).

Hornung et al. demonstrated that mice with obesity had a delayed onset of body mass loss compared to non-obese mice, and they did not recover to baseline body mass until 21 dpi (34). Likewise, other studies have shown delayed mononuclear cell infiltration in the lungs, altered T cell responses, and a late rise in cytokine and chemokine responses in mice with obesity (35, 36). In our study, both males and females with obesity lost significantly greater body mass compared to their non-obese controls, and did not recover to the baseline body mass even at 21 dpi, indicating the obesity-associated disease severity in both sexes. This reflects sustained inflammatory response and immune activation in mice with obesity, which may be responsible for the lack of weight recovery in mice with obesity. When comparing males and females with obesity, females lost greater body mass and temperature than males, and 25% of the females even reached the humane endpoint, indicating sex differences following a low-dose IAV infection.

The sex difference in the low-dose IAV infection during obesity was not directly reflected by differences in lung virus replication. In both sexes, lung pathology peaked at 10 dpi and decreased at 21 dpi, but the residual lung inflammation was relatively higher in females with obesity. Females with obesity maintained robust induction of innate and inflammatory cytokines, including IFN-α, TNF-α, and GRO-α, even at 21 dpi, a timepoint when inflammation typically resolves during IAV infection. Compared to the males, females with obesity had elevated levels of common gamma chain cytokines (i.e., IL-2, IL-4, IL-7, and IL-9), which are involved in lymphocyte activation, differentiation, and survival (37). In addition, females maintained higher levels of IL-10 family cytokines (i.e., IL-10 and IL-19) which are generally associated with immunoregulatory and anti-inflammatory signaling, as well IL-12 family cytokines (i.e., IL-12p70, IL-23, and IL-27), which promote Th1- and Th17-associated immune responses (38). These observations suggest that females with obesity exhibit persistent inflammation even at 21 dpi, reflecting inefficient inflammation resolution and a more pronounced dysregulation of inflammatory and adaptive immune pathways compared to males with obesity (39).

Females, irrespective of obesity, suffered from more severe disease following IAV infection compared to males. Following low-dose infection, females with or without obesity had similar levels of body mass loss at the peak of the disease progression. However, while non-obese females recovered from the severe disease, females with obesity could not. Interestingly, virus titer trended higher in non-obese females than in females with obesity at 3 dpi, suggesting the differences in disease severity to be mediated by host responses rather than lung virus titers alone. It is better explained by the delayed but persistent dysregulated inflammatory response in females with obesity than in non-obese females. Non-obese females displayed the typical course of IAV infection, with robust inflammatory responses by 3 dpi that were sustained or peaked at 10 dpi and resolved by 21 dpi. In contrast, females with obesity showed delayed inflammatory responses; inflammation was less pronounced at 3 dpi, peaked at 10 dpi, and remained elevated at 21 dpi. This delayed response in females with obesity was supported by lower frequencies of myeloid and lymphoid cell infiltration and lower lung pathology at 3 dpi, along with a shift from lower cytokine and chemokine induction at 3 dpi to higher induction at 21 dpi. In accordance, Smith et al. also showed reduced interstitial macrophages and dendritic cells in IAV-infected mice with obesity as compared to non-obese mice at 3 dpi (35). Reduced number and function of dendritic cells is also observed in humans with obesity (40). The non-responder females, treated with HFD but did not gain body mass to be defined as having obesity, also had disease severity, lung virus titers, and inflammatory changes comparable to females with obesity. These findings suggest that the metabolic alterations induced by long-term HFD treatment may drive risk independently of overt obesity. Therefore, future studies should compare metabolic phenotypes before and after IAV infection between non-responder and responder females. Following recovery from IAV infection, antibody responses are shown to be higher in non-obese females than in non-obese males, both in serum as well as in bronchoalveolar lavage (BAL) fluid (41). It is associated with a greater number of antibody-secreting B cells, T-helper cells, and germinal center B cells in the lungs of females than in males (41). We also observed greater systemic and mucosal antibody responses in non-obese females compared to non-obese males. Further, this study suggests that female sex drives higher antibody responses following recovery from IAV infection, even during obesity, as demonstrated by higher IgG and neutralizing antibodies in the plasma and production of mucosal antibodies by a larger number of female mice than male mice with obesity. This observation most likely reflects the female sex-associated robust induction of B-cell responses and humoral immunity, mediated by hormonal and chromosomal factors (22, 42, 43), which needs further exploration during obesity. It also suggests that greater disease severity in females is likely caused by a dysregulated innate immune response rather than a failure of humoral priming. Future studies should investigate whether a higher antibody response in females than in males with obesity confers better protection during IAV reinfection.

There are certain limitations in our study. We used only the mouse-adapted 2009 H1N1 IAV strain, and the results might vary with different strains and subtypes of influenza viruses. Immune cell phenotyping was performed only at acute infection (i.e., 3 dpi), which limited the evaluation of temporal changes in the immune cell environment during disease progression. As only one half of the lungs was used for flow cytometry, absolute cell numbers could not be determined to reflect the differences in total cellularity across the groups. The females with obesity were smaller and developed less severe hyperglycemia than males with obesity. This might overestimate the interpretation of the biological sex difference. Estrous cycle variation in females could also modulate inflammatory responses, and it could not be controlled in this model. The mouse model is inbred and may not entirely recapitulate the genetic diversity existing in humans. Though we showed the phenotypic difference, this study does not explain the underlying mechanisms, such as the role of specific sex steroids, adipokines, cytokines, and genetic differences between males and females with obesity that require further exploration in the future.

In sum, this study provides a temporal analysis of IAV pathogenesis between male and female mice, with and without obesity, following both the high- and low-dose IAV infection. Biological sex difference was observed in influenza severity, where females, irrespective of obesity status, developed more severe disease than males. Likewise, obesity-associated increase in disease severity was also observed in both male and female DIO mice, especially after the low-dose IAV infection. After the low-dose IAV infection, females with obesity exhibited the greatest disease severity, likely driven by dysregulated inflammatory responses and disproportionate immune cell infiltration. These findings emphasize the need to account for biological sex differences in IAV pathogenesis studies and in the interpretation of preclinical, clinical, and epidemiological data in populations with obesity. It will be critical to better understand the mechanisms of immune dysfunction during virus infection in obesity and to develop safer and more effective preventive and therapeutic strategies.

## Data Availability

The original contributions presented in the study are included in the article/**Supplementary Material**. Further inquiries can be directed to the corresponding author.
