# Supplementary material for: Biological sex differences after high and low doses of influenza A virus infection during obesity
Source: Front Immunol. 2026 Apr 10;17:1788970. doi: 10.3389/fimmu.2026.1788970 (PMC13106206; doi:10.3389/fimmu.2026.1788970)
Supplement: Supplementary file 1 [file DataSheet1.pdf]

Biological sex differences after high and low doses of influenza A virus infection during obesity

Supplementary Material

Supplementary Figure 1.

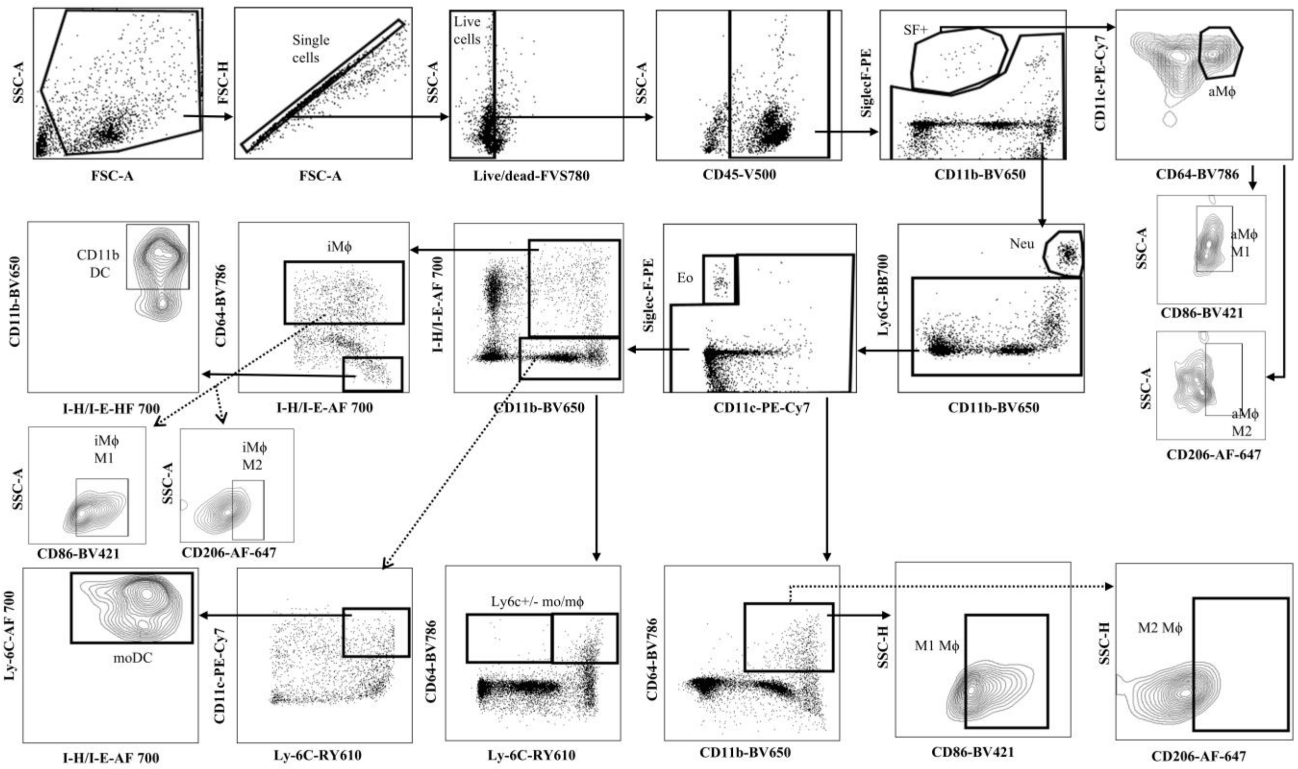

Gating strategy for myeloid cells in the lungs. Male and female C57BL/6J mice, with or without obesity, were infected with a low dose (i.e., $10^{1.5}$ $TCID_{50}$) of the 2009 pandemic H1N1 virus. After 3 days post-infection (dpi), mice were euthanized, cells were prepared from the lungs, stained with different antibody markers, and acquired on a flow cytometer to determine the frequencies of different myeloid cell populations. The representative gating strategy is illustrated using data from one mouse. Total cells were gated, followed by single cells, and live cells using the live/dead staining dye. $CD45^{+}$ cells were then selected and alveolar macrophages ($CD45^{+}SiglecF^{+}CD11b^{-/lo}CD11c^{+}CD64^{+}$), neutrophils ($CD45^{+}CD11b^{+}Ly6G^{+}$), eosinophils ($CD45^{+}SiglecF^{+}CD11c^{-}$), interstitial macrophages ($CD45^{+}CD11b^{+}MHC\ II^{+}CD64^{int/hi}$), $CD11b^{+}$ DC ($CD45^{+}CD11b^{+}MHC\ II^{+}CD64^{-}$), M1 macrophages ($CD45^{+}CD64^{+}CD11b^{+}CD86^{+}$), M2 macrophages ($CD45^{+}CD64^{+}CD11b^{+}CD206^{+}$), inflammatory ($Ly6c^{+}$) monocytes/macrophages ($CD45^{+}CD64^{+}CD11b^{+}Ly6c^{+}$), and monocyte-derived dendritic cells (moDCs) ($CD45^{+}CD11b^{+}MHCII^{+/-}CD11c^{+}Ly6c^{+}$) were gated.

Supplementary Figure 2.

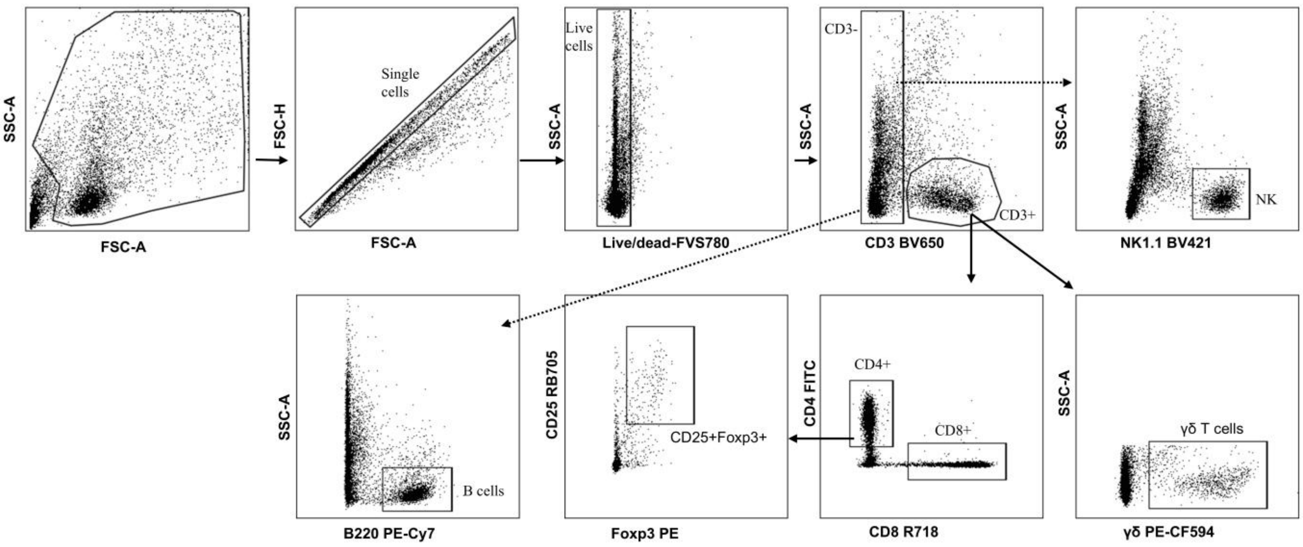

Gating strategy for lymphoid cells in the lungs. Male and female C57BL/6J mice, with or without obesity, were infected with a low dose (i.e., $10^{1.5}$ $TCID_{50}$) of the 2009 pandemic H1N1 virus. After 3 days post-infection (dpi), mice were euthanized, cells were prepared from the lungs, stained with different antibody markers, and acquired on a flow cytometer to determine the frequencies of different lymphoid cell populations. The representative gating strategy is illustrated using data from one mouse. Total cells were gated, followed by single cells, and live cells using the live/dead staining dye. $CD3^+$ cells were gated followed by gating for T helper ($CD3^+CD4^+$), $CD8^+$ T cells ($CD3^+CD8^+$), and gamma-delta T cells ($CD3^+\gamma\delta^+$). $CD4^+$ cells were further gated to determine the frequency of regulatory T (Treg) cells ($CD3^+CD4^+CD25^+Foxp3^+$). $CD3^-$ cells were used to determine the frequencies of NK cells ($CD3^-NK1.1^+$) and B cells ($CD3^-B220^+$).

Supplementary Figure 3.

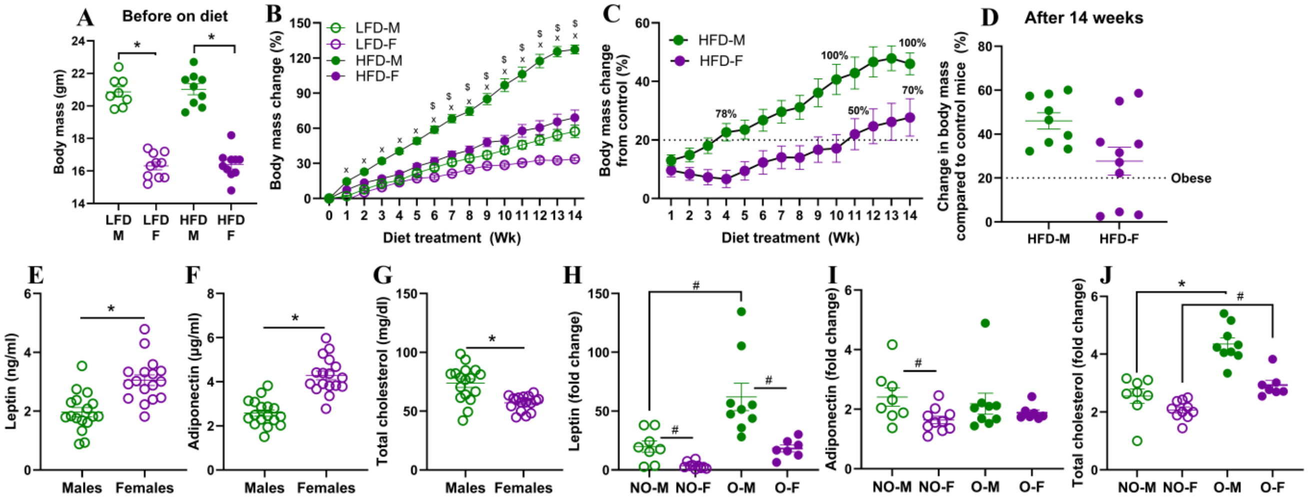

Body mass and blood parameters after diet treatment in male and female mice. C57BL/6J male and female mice of age 5-6 weeks were treated with a low-fat diet (LFD) or a high-fat diet (HFD) for 14 weeks. (A) Body mass before the start of the diet treatment; (B) percentage change in body mass following the diet treatment; (C) percentage change in body mass from age- and sex-matched controls over time, and (D) at the 14th week are shown. Blood biomarkers were also measured before diet treatment and after the 14th week of diet treatment. The concentrations of (E) leptin, (F) adiponectin, and (G) total cholesterol before diet treatment, and (H-J) fold changes in these biomarkers by the end of the 14th week, respectively, are shown. Data is shown as mean ± standard error of the mean (SEM) (n=7-10/group). Statistical comparisons were made using one-way ANOVA and Tukey's test or Kruskal-Wallis and Dunn's post-hoc tests. Data in B were analyzed using two-way repeated measures ANOVA (mixed model) followed by Tukey's multiple comparisons. An asterisk (*) indicates a statistically significant difference ($p<0.05$) while a hash ($^{\#}$) indicates a trend ($0.05 \leq p \leq 0.1$). In Figure B, 'x' and '$' represent significant differences between non-obese (or LFD) versus obese (or HFD) males and females, respectively. Abbreviations: LFD-M, males on low-fat diet; LFD-F, females on low-fat diet; HFD-M, males on high-fat diet; HFD-F, females on high-fat diet; NO-M, non-obese males; NO-F, non-obese females; O-M, males with obesity; and O-F, females with obesity.

Supplementary Figure 4.

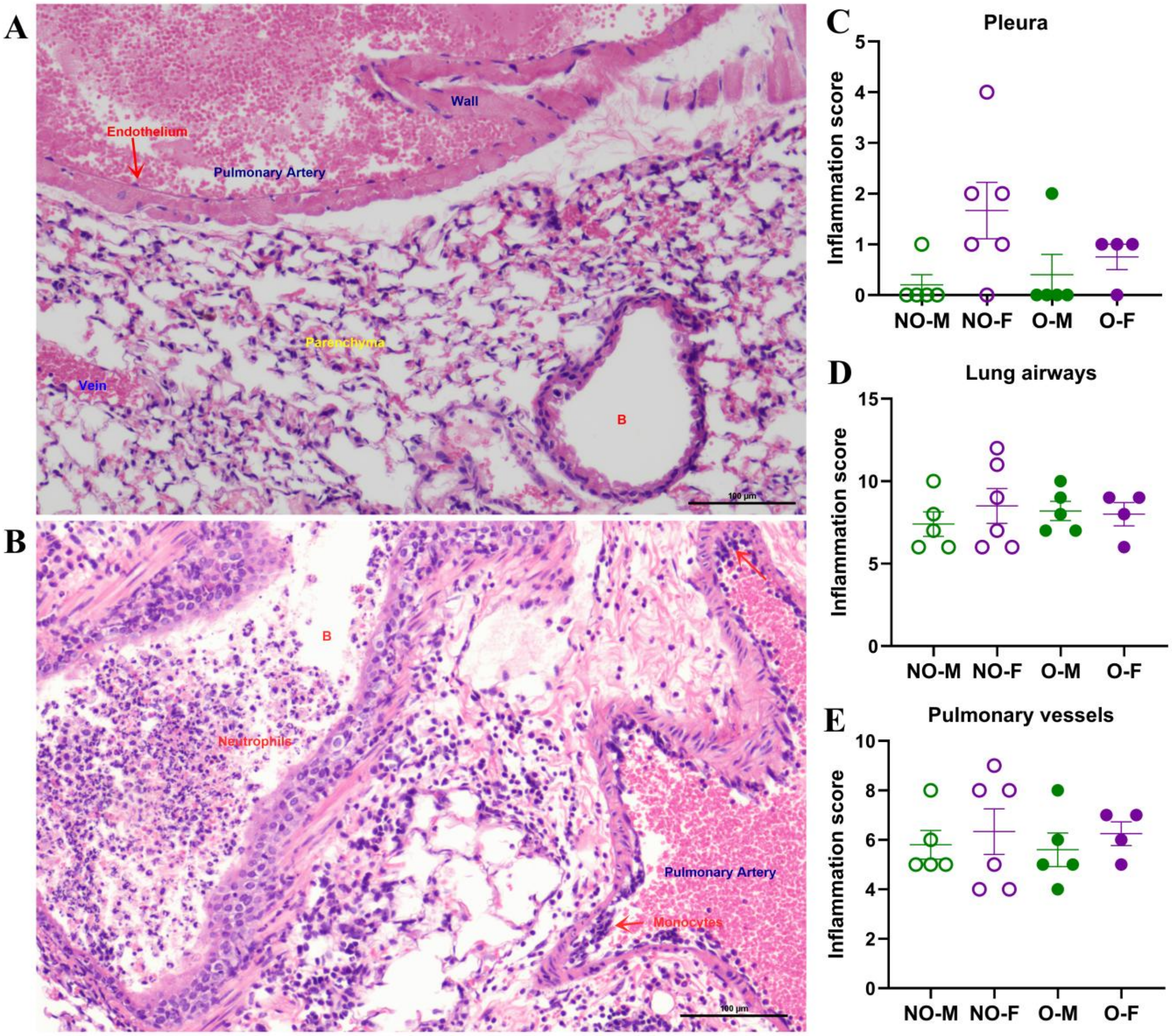

Inflammatory changes in the lungs after a high-dose infection. Males and females, with or without obesity, were infected with a high dose (i.e., 10^3 $TCID_{50}$) of the 2009 pandemic IAV, and at 3 days post-infection, lungs were fixed and used for histopathological analysis using H&E staining. Representative lung images from (A) medium-inoculated and (B) virus-inoculated mice are shown. Inflammatory changes in the (C) pleura, (D) lung airways, and (E) pulmonary vessels are compared. Data is shown as mean ± standard error of the mean (SEM) (n=5-6/group). Statistical comparisons were made using one-way ANOVA and Tukey's post-hoc test or Kruskal-Wallis and Dunn's multiple comparisons. Abbreviations: NO-M, non-obese males; NO-F, non-obese females; O-M, males with obesity; and O-F, females with obesity.

Supplementary Figure 5.

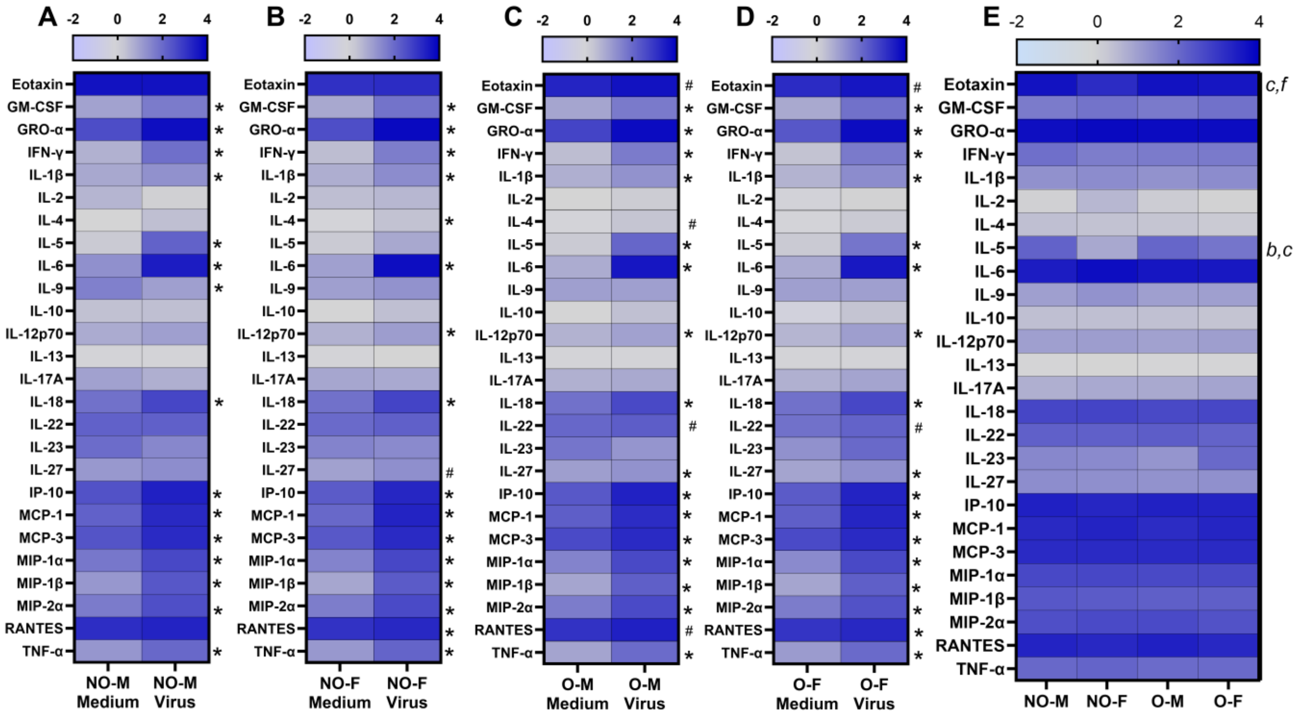

Cytokines and chemokines in the lungs following high-dose IAV infection. Males and females, with or without obesity, were infected with a high dose (i.e., 10^3 $TCID_{50}$) of the 2009 pandemic H1N1 virus or vehicle (i.e., medium) only. At 3 days post-infection (dpi), mice were euthanized, and cytokine and chemokine responses were measured in the lung homogenates. Comparison of absolute concentrations of log_{10}-transformed cytokine and chemokine responses between medium-inoculated and virus-infected (A) non-obese males (NO-M), (B) non-obese females (NO-F), (C) males with obesity (O-M), and (D) females with obesity (O-F) is shown (n=3-6/group). Likewise, (E) comparisons of cytokines and chemokines among virus-infected NO-M, NO-F, O-M, and O-F are shown. Statistical analysis was carried out by unpaired t-tests, followed by Holm-Sidak correction for multiple comparisons, and one-way ANOVA or Kruskal-Wallis test, followed by Benjamini and Hochberg multiple comparisons. Data were considered statistically significant at $p<0.05$ and having a trend at $0.05 \leq p \leq 0.1$. Figures A-D: an asterisk (*) represents a significant difference, and a hash represents a trend. In Figure E, a and e; b and f; c and g; and d and h represented a significant difference or a trend between NO-M versus O-M; NO-F versus O-F; NO-M versus NO-F; and O-M versus O-F, respectively.

Supplementary Figure 6.

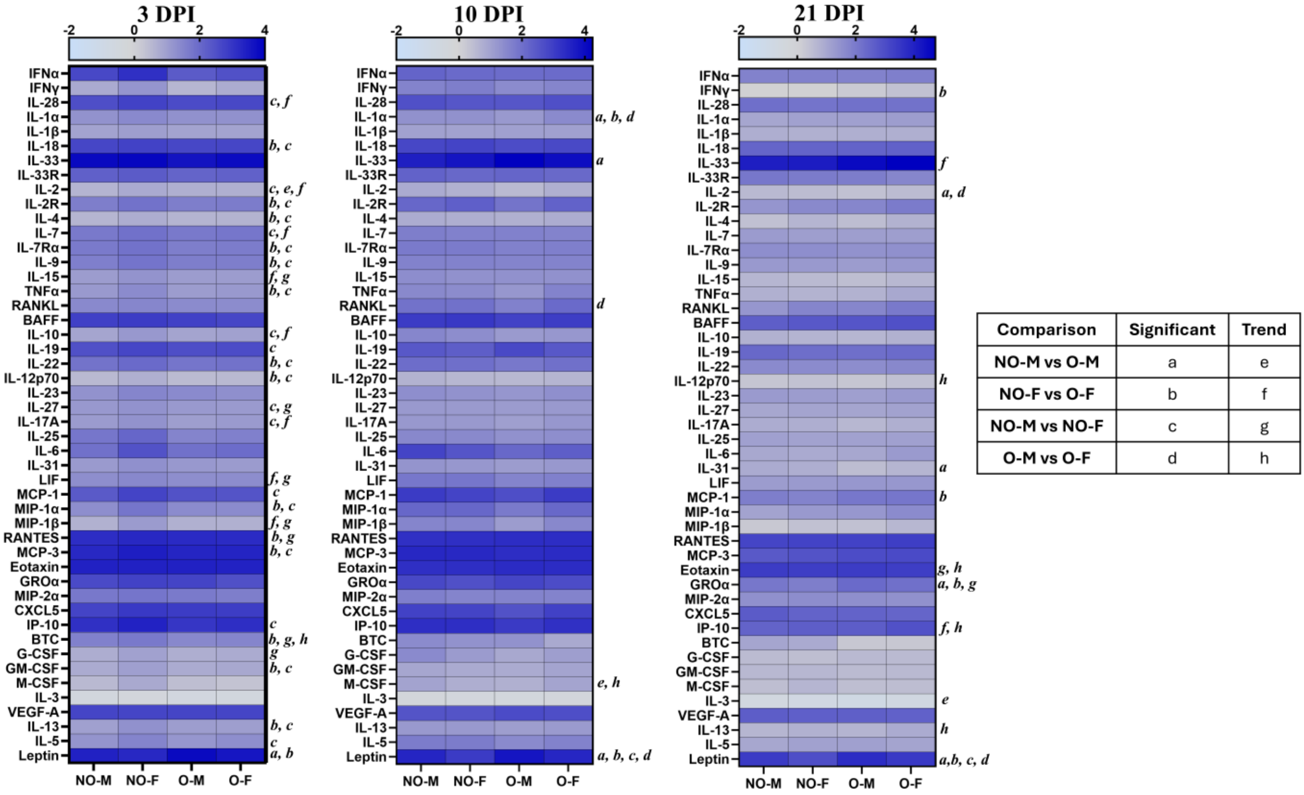

Comparison	Significant	Trend
NO-M vs O-M	a	e
NO-F vs O-F	b	f
NO-M vs NO-F	c	g
O-M vs O-F	d	h

Comparison of absolute concentrations of cytokine and chemokine responses in the lungs following low-dose IAV infection. After infection with a low dose (i.e., $10^{1.5}$ $TCID_{50}$) of the 2009 pandemic H1N1 IAV, subsets of mice were euthanized at 3-, 10-, and 21-days post-infection (dpi), lungs were collected, and various cytokines and chemokines were measured. Absolute concentrations of cytokines and chemokines (log_{10}-transformed) among virus-infected mice euthanized at 3 dpi, 10 dpi, and 21 dpi are compared. Statistical comparison was made using one-way ANOVA or Kruskal-Wallis test, followed by Benjamini and Hochberg multiple comparisons. Data were considered statistically significant at $p<0.05$ and having a trend at $0.05 \leq p \leq 0.1$. Letters a and e; b and f; c and g; and d and h represented a significant difference or a trend between non-obese males (NO-M) versus males with obesity (O-M); non-obese females (NO-F) versus females with obesity (O-F); non-obese males (NO-M) versus non-obese females (NO-F); and males with obesity (O-M) versus females with obesity (O-F), respectively.

Supplementary Figure 7.

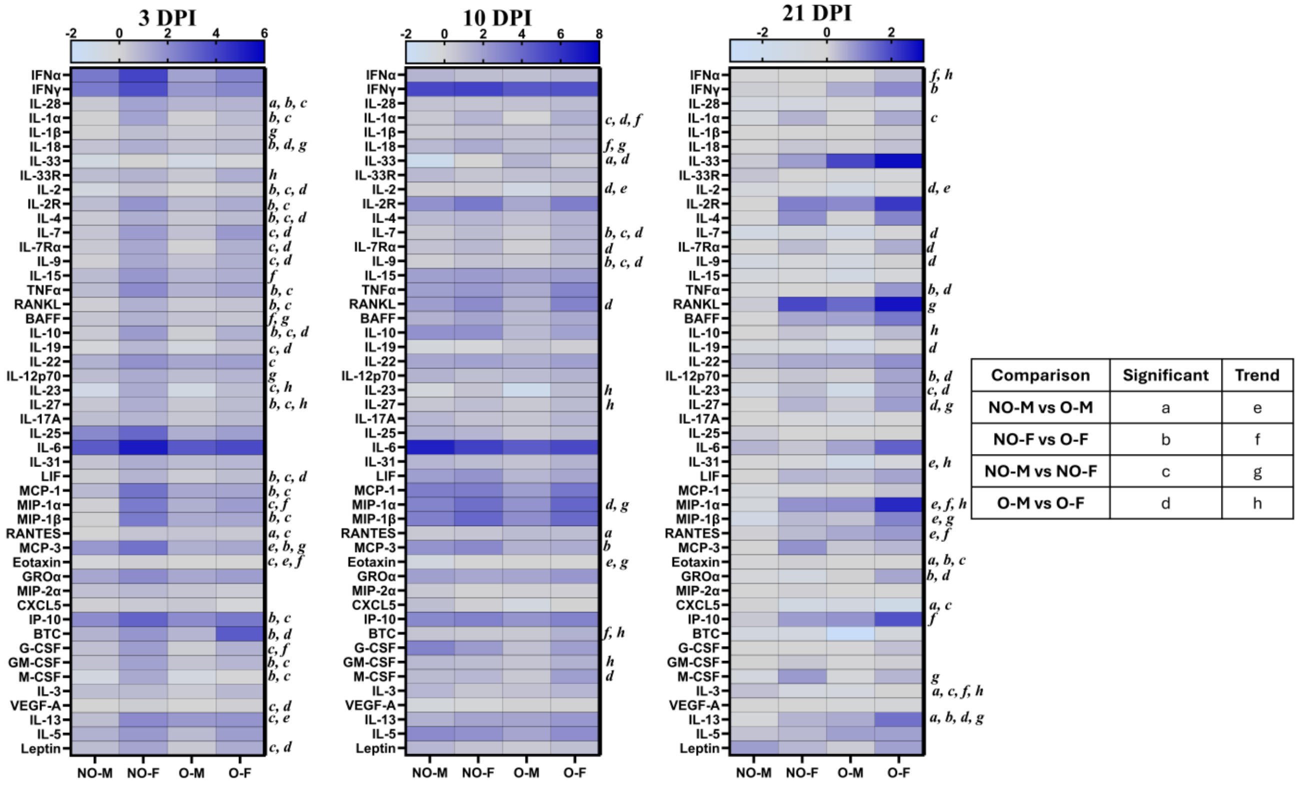

Comparison	Significant	Trend
NO-M vs O-M	a	e
NO-F vs O-F	b	f
NO-M vs NO-F	c	g
O-M vs O-F	d	h

Comparison of fold changes of cytokine and chemokine responses in the lungs following low-dose IAV infection. After infection with a low dose (i.e., $10^{1.5}$ $TCID_{50}$) of the 2009 pandemic H1N1 IAV, subsets of mice were euthanized at 3-, 10-, and 21-days post-infection (dpi), lungs were collected, and various cytokines and chemokines were measured. Fold changes in virus-infected mice at 3-, 10-, and 21-dpi were calculated based on cytokine and chemokine responses in medium-inoculated controls of respective groups euthanized at 3 dpi. Statistical comparison was made using one-way ANOVA or Kruskal-Wallis test, followed by Benjamini and Hochberg multiple comparisons. Data were considered statistically significant at $p<0.05$ and having a trend at $0.05 \leq p \leq 0.1$. For graphical presentation, fold changes were $\log_2$-transformed. Letters a and e; b and f; c and g; and d and h represented a significant difference or a trend between non-obese males (NO-M) versus males with obesity (O-M); non-obese females (NO-F) versus females with obesity (O-F); non-obese males (NO-M) versus non-obese females (NO-F); and males with obesity (O-M) versus females with obesity (O-F), respectively.

Supplementary Figure 8.

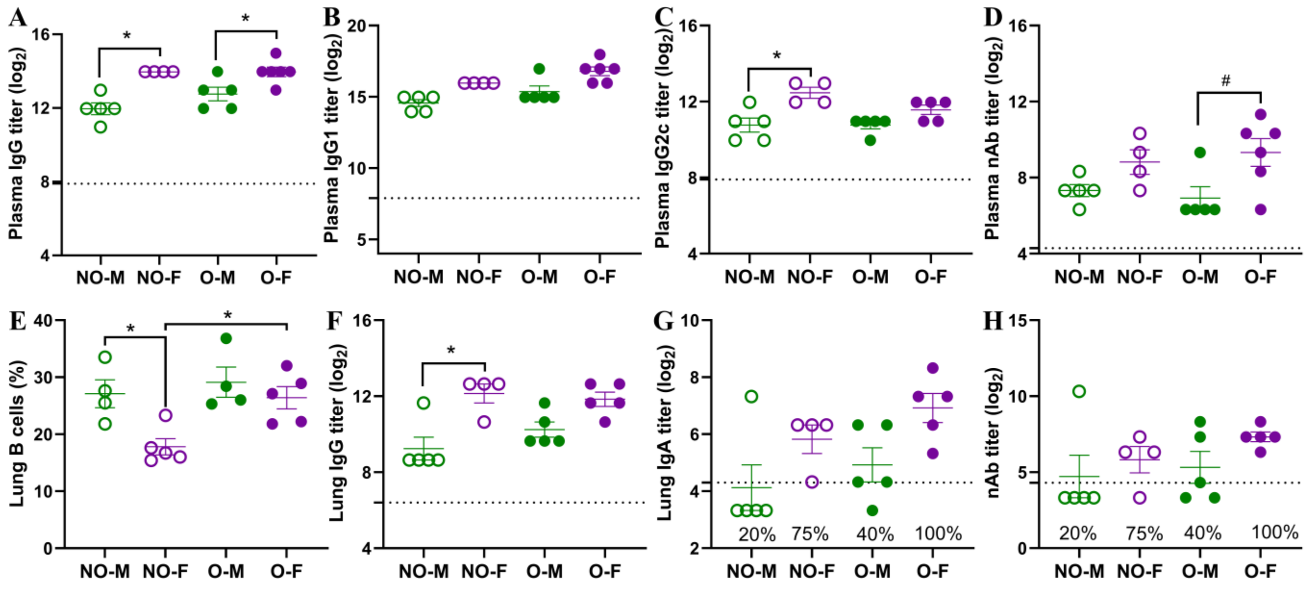

Antibody responses in plasma and lung homogenates following low-dose IAV infection. After infection with a low dose (i.e., $10^{1.5}$ $TCID_{50}$) of the 2009 pandemic H1N1 IAV, mice were followed for morbidity measurements up to 21 dpi. At 21 dpi, after euthanization, plasma samples and lung homogenates were prepared to measure systemic and mucosal antibody responses. Plasma levels of (A) IgG, (B) IgG1, (C) IgG2c, and (D) virus-neutralizing antibody (nAb) titers are compared. (E) The frequency of B-cells in the lungs, as measured by flow cytometry in the subsets of mice euthanized at 3 dpi is shown. Likewise, pulmonary (F) IgG, (G) IgA, and (H) nAb titers are compared. Data is shown as mean ± standard error of the mean (SEM) (n=4-6/group). Percentage (%) values indicate animals that had antibody responses higher than the limit of detection (dashed line). Statistical comparisons were made using one-way ANOVA or Kruskal-Wallis test followed by Tukey's or Dunn's multiple comparisons, respectively. An asterisk (*) indicates a statistically significant difference ($p<0.05$) while a hash (#) indicates a trend ($0.05 \leq p \leq 0.1$). Abbreviations: NO-M, non-obese males; NO-F, non-obese females; O-M, males with obesity; and O-F, females with obesity.
